# Green solvent free epoxidation of olefins by a heterogenised hydrazone-dioxidotungsten(vi) coordination compound†

**DOI:** 10.1039/d1ra09217k

**Published:** 2022-02-09

**Authors:** Neda Heydari, Rahman Bikas, Maryam Shaterian, Tadeusz Lis

**Affiliations:** Department of Chemistry, Faculty of Science, University of Zanjan 45371-38791 Zanjan Iran; Department of Chemistry, Faculty of Science, Imam Khomeini International University 34148-96818 Qazvin Iran bikas@sci.ikiu.ac.ir bikas_r@yahoo.com; Faculty of Chemistry, University of Wroclaw Joliot-Curie 14 Wroclaw 50-383 Poland

## Abstract

A new mononuclear tungsten coordination compound, [WO_2_L(CH_3_OH)] (1), was synthesized by the reaction of WCl_6_ and H_2_L (H_2_L = (*E*)-4-amino-*N*′-(5-bromo-2-hydroxybenzylidene)benzohydrazide) in methanol. Both the H_2_L and compound 1 were characterized by elemental analysis and UV-Vis, FT-IR and NMR spectroscopic methods. The molecular structure of compound 1 was also determined by single crystal X-ray analysis which confirmed the compound is a mononuclear coordination compound of *cis*-dioxidotungsten(vi) containing a free amine functionality on the ligand. Compound 1 was supported on propionyl chloride-functionalized silica gel by amidification reaction to obtain a heterogeneous catalyst. The obtained heterogeneous catalyst was characterized by FT-IR spectroscopy, thermal gravimetric analysis (TGA), diffuse-reflectance spectroscopy (DRS), X-ray diffraction analysis (XRD), energy-dispersive X-ray spectroscopy (EDX), X-ray photoelectron spectroscopy (XPS) and scanning electron microscopy (SEM) and its catalytic activity was investigated in the epoxidation of olefins with hydrogen peroxide under solvent free conditions. The catalyst was successfully recovered several times and the recovered catalyst was also characterized by various methods including FT-IR, DRS, TGA, SEM and EDX analyses. The results indicated this heterogeneous catalytic system is an effective and selective catalyst for epoxidation of olefins and can be reused several times without significant change in its catalytic activity.

## Abstract 2

In this study the solvent free catalytic oxidation of olefins by a new silica supported hydrazone-dioxidotungsten(vi) coordination compound is investigated.

## Graphical abstract

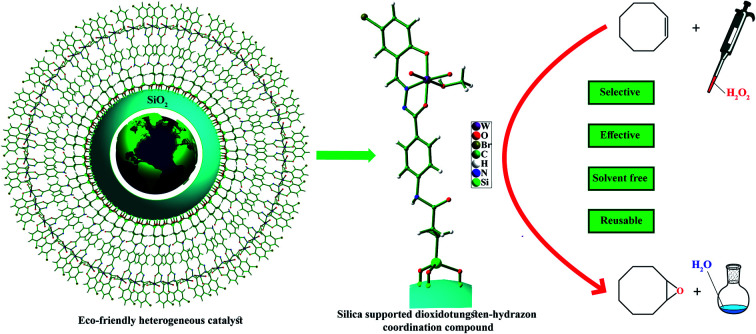

## Introduction

Epoxides are very important intermediates in the chemical science and pharmaceutical industries because they are key materials for the synthesis of wide range of organic compounds.^1^ Nowadays, the epoxidation of olefins using various methods has become an important chemical reaction for the production of epoxides because this process can convert relatively cheap and available olefins into expensive epoxide intermediates.^2^ Catalytic epoxidation of olefins in the presence of green oxidants is one of the most important methods for epoxidation of olefins.^3^ Due to this, various catalysts based on transition metal coordination compounds have been used for this transformation and among them high valent metal-oxido catalysts (such as vanadium, molybdenum and tungsten) have attracted considerable attention by considering their high activity in catalytic oxidation reactions.^4^ Although there are many reports on the catalytic epoxidation of olefins with oxido-vanadium^5^ and oxido-molybdenum^6^ coordination compounds, considering literature indicates there are limited reports for epoxidation reaction by oxido-tungsten coordination compounds while it has been observed that they are also very useful and effective catalysts for epoxidation of olefins. This matter maybe is related to the low number of successful methods for the synthesis of tungsten coordination compounds. The use of tungsten Schiff base coordination compounds as a catalyst for epoxidation of olefins has significant advantages such as commercial availability, high stability and high efficiency.^7^

Considering literature indicates some of the transition metal coordination compounds with hydrazone ligands are effective, selective and stable catalysts for oxidation reactions.^8^ Nevertheless, these compounds usually work homogeneously and the problems related to homogeneous catalysts (like separation and purification processes) have led to considerable limitations for the use of this type of catalysts. In recent years, heterogenising processes like supporting coordination compounds on suitable surfaces have been developed to produce heterogeneous catalysts based on transition metal coordination compounds.^9^ It should be noted that several factors like solvents, structure of ligand, substitutions on the ligands, co-ligands, type of the metal salt and reaction condition (pH, temperature, pressure, and reaction atmosphere, *etc.*) have high influence on coordination compounds and slight change in the mentioned parameters can considerably change the structure and nuclearity of the product,^10^ Due to this, using strong and high accurate structural analyses like single crystal X-ray analysis are urgent to find the real structure of coordination compounds and determine the coordination environments around metal ions. Coordination environment and the structure of coordination compounds have considerable influence on their activity in various applications.^11^ Thus, the problems such as difficulties in identification of catalytically active species as well as problems related to determining the exact coordination environment around the metal ions are some of the challenging issues in the characterization of heterogeneous system based on coordination compounds.^12^ Preparing coordination compounds with suitable functional groups on their structure to react with surfaces and determining their structures by single crystal X-ray analysis before supporting process is one of the suitable strategies to solve part of this problem. In such a case, from both coordination chemistry and solid state chemistry considerations suitable and sufficient information can be provided about the composition of heterogeneous catalysts based on coordination compounds. Moreover, prediction of the structure of catalytically active species on the surface of heterogeneous catalysts to apply specific changes and increase their catalytic performance can be achievable. This matter can also connect these two fields to each other and overcome to the scientific challenges regarding the structure of coordination compounds in heterogeneous catalytic systems. Nevertheless, this requires effective and professional synthetic strategies to design and prepare specific ligands and coordination compounds. Hydrazones have high diversity and by applying special synthetic strategies variety of appropriate functional groups can be placed on them to bind on the heterogeneous surfaces.

Using the least solvents and materials in synthetic and catalytic systems is one of the major goals of green chemistry.^13^ Therefore, designing solvent-free catalytic systems and using green oxidants (like molecular oxygen or hydrogen peroxide) in catalytic epoxidation systems are very important issues to get closer to the goals of green chemistry.^14^

In this project, we synthesized a new hydrazone tungsten coordination compound by using special synthetic strategies, which has an amine functional group that can be supported on propionyl chloride-functionalized silica gel to form a stable heterogeneous catalyst. The coordination compound (1) was synthesized by using tridentate ONO-donor hydrazone ligand containing a free amine functionality. Then, it was heterogenised by supporting on the surface of propionyl-chloride functionalized silica gel. The structure of free coordination compound was characterized by single crystal X-ray analysis and a mixture of spectroscopic methods (FT-IR, NMR, UV-Vis, DRS), TGA, XRD, SEM, EDX and XPS analyses were employed to characterize its heterogenised system. The heterogeneous catalyst has been investigated in the epoxidation of olefins and the results showed it has excellent activity and selectivity in the epoxidation of olefins. Moreover, the catalyst is recoverable for several times with no significant loss in its catalytic activity.

## Results and discussion

### Synthesis and characterization

**Scheme 1:**
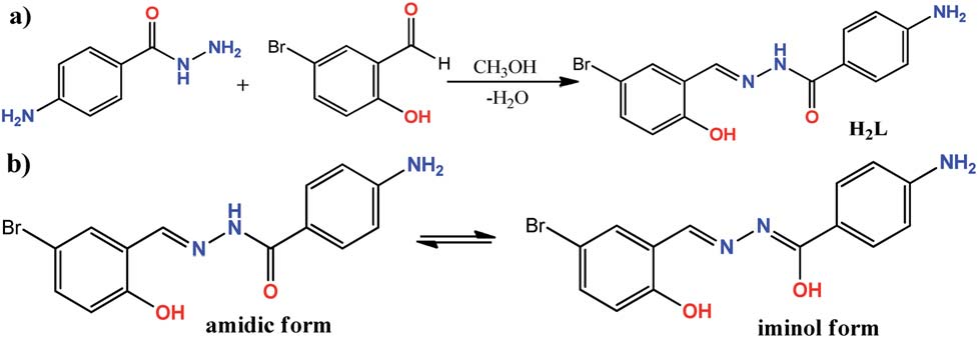
(a) The reaction for the synthesis of H_2_L; (b) amidic and iminol forms of H_2_L; (c) chemical equation for the formation of compound 1.

The ONO donor hydrazone ligand, (*E*)-4-amino-*N*′-(5-bromo-2-hydroxybenzylidene)benzohydrazide, was synthesized by the condensation of 4-aminobenzoic hydrazide with 5-bromo-2-hydroxybenzaldehyde in 1 : 1 molar ratio under reflux condition in methanol (see Scheme 1). Although there are two primary amine functionalities in the structure of 4-aminobenzoic hydrazide, only the –NH_2_ group of hydrazide moiety (–CO–NH–NH_2_) is involved in Schiff base condensation and the Ph–NH_2_ group is remained intact during the synthesis of H_2_L. This matter is related to low activity of Ph–NH_2_ group in comparison to R–NH–NH_2_ moiety. However, it should be noted that the excess amount of aldehydes can interact with Ph–NH_2_ group to form a byproduct. Therefore, controlling the equimolar ratio of reagents should be considered and the weight of the reagents should be calculated with high care. The formation of H_2_L as the sole product in high purity was confirmed by elemental analysis and various spectroscopic methods. In the FT-IR spectrum of H_2_L (Fig. S1†), the band at 1603 cm^−1^ is due to imine C=N group and the band at 1625 cm^−1^ can be attributed to the amidic C=O.^15^ The broad peak at 3471 cm^−1^ is due to the phenolic OH group and the bands at 3214 and 3354 cm^−1^ are due to the NH and NH_2_ groups.^16^ The bands of NH, NH_2_ and OH groups are broad and overlapped which confirm they are involved in hydrogen bond interactions. In the ^1^H NMR spectrum of H_2_L (see Fig. S2†) the OH proton is observed at *δ* = 11.55 ppm. The signal for protons of NH_2_ group is observed at *δ* = 5.85 ppm and the proton of amidic NH is observed as a singlet peak at *δ* = 11.86 ppm. Upon addition of D_2_O the intensities of these signals was significantly decreased (see Fig. S3†). A singlet peak at *δ* = 8.52 ppm is related to the imine (–CH=N) group and the aromatic protons of both rings are observed in the range of *δ* = 6.59–7.71 ppm.^17^ There are 12 unique carbon atoms in the molecules, which give 12 different peaks in the ^13^C NMR spectrum (see Fig. S4†). The peaks observed at *δ* = 163.3, 156.8, 153.1, 145.0 and 110.8 ppm in the ^13^C NMR spectrum of H_2_L are due to amidic C=O, phenolic C–O, aromatic C–NH_2_, imine C=N and aromatic C–Br groups, respectively.

H_2_L was employed as ligand in preparing tungsten coordination compound by the reaction of ligand with WCl_6_ in methanol (Scheme 1). Compound 1 was obtained as orange single crystals during one week which were characterized by elemental analysis, several spectroscopic methods and single crystal X-ray analysis. The results of the analyses confirmed the formation of a W(vi) coordination compound with general formula of [WO_2_L(CH_3_OH)] (1) according to the equation shown in Scheme 1c. The reaction for the formation of compound 1 was slow and the results indicated all of the chloride anions in the starting WCl_6_ have been eliminated and the W(vi) ion is converted to (WO_2_)^2+^ unit during the formation of compound 1. Since there is not any oxidation–reduction reaction in this process, it was clear that the water molecules of methanol solvent were the source of oxygen atoms in the formation of (WO_2_)^2+^ unit. This matter was also confirmed by the fact that the rate of reaction is considerably depended on the amount of water dissolved in methanol solvent. When the process was carried out in dried methanol solvent, the reaction did not take place while in the presence of higher amount of water (5, 10 and 20 v/v%), the rate of the reaction was considerably increased. By considering the results of this reaction, we performed the reaction of H_2_L with WO_3_ as the source of W(vi) ion in methanol but surprisingly, there was not any product in this reaction. This matter indicates the use of WCl_6_ as the source of W(vi) ion and also the presence of water molecules are important requirements for the formation of compound 1. In the FT-IR spectrum of compound 1 (Fig. S5†), the strong band observed at 1605 cm^−1^ can be attributed to the C=N stretching frequency of the coordinated ligand.^18^ The elimination of amidic C=O band confirms the coordination of ligand in iminol form^19^ and the peak at 1627 cm^−1^ can be assigned to –C=N–N=C– which is created in iminol form. The broad peaks at 3347 and 3244 cm^−1^ are due to the NH_2_ group and the broad band at about 3440 cm^−1^ can be assigned to the OH group of the methanol molecule which is also overlapped with the bands of NH_2_ group. The bands at 947 and 902 cm^−1^ are attributed to the presence of *cis*-WO_2_ group in the structure of compound 1.^20^ The ^1^H and ^13^C NMR spectra of compound 1 (recorded in DMSO-d_6_) are shown in Fig. S6 and S7,† respectively. In the ^1^H NMR spectrum, the broad peak at *δ* = 3.39 (OH proton of methanol) and the singlet peak at *δ* = 3.14 ppm (–CH_3_ of CH_3_OH) confirm the presence of methanol in the structure of compound 1. The signal for NH_2_ is observed at *δ* = 6.01 ppm. Upon addition of D_2_O, the intensity of the OH and NH_2_ signals was significantly decreased (Fig. S8†). The imine hydrogen is observed at *δ* = 8.70 ppm as a singlet peak and the shift to higher frequencies respect to the free ligand indicates the coordination of imine nitrogen to the metal core. Aromatic protons are located in the range of 6.6–7.9 ppm. The presence of 13 unique peaks in the ^13^C NMR spectrum of compound 1 (Fig. S7†) is in agreement with the structure of this compound. The signal of methyl group of coordinated methanol is located at *δ* = 49.1 ppm. The peaks of iminolic C–O, phenolic C–O, aromatic C–NH_2_, imine C=N and aromatic C–Br groups were observed at *δ* = 170.0, 157.4, 153.6, 158.1 and 113.0 ppm, respectively.^21^ The shift of the position of these peaks to higher frequencies respect to the free ligand confirms the coordination of the ligand to the W(vi) ion.

TGA diagram of compound 1 exhibits three steps weight losses (Fig. S9†). The first step of the weight loss from 100 °C to 250 °C, corresponds to the removal of coordinated methanol molecules. This indicates the W–methanol bond is weaker than other groups but it considerably stabilizes the methanol molecule in the structure of compound 1. The observed weight loss of 8.20% is close to the calculated value (5.52%). The removal of organic ligand was occurred in two steps (350–530 °C and 550–950 °C) with the main weight loss in the range of 350–530 °C. In these two steps, the observed weight loss of 55.15% is very close to the calculated value (57.61%). By considering the total weight loss of 63.40%, the remaining weight is equal to 36.60% of the starting mass. Therefore, the final product of the thermal decomposition of compound 1 is estimated to be WO_3_ (calculate 39.97%, observed 36.60%).

**Fig. 1:**
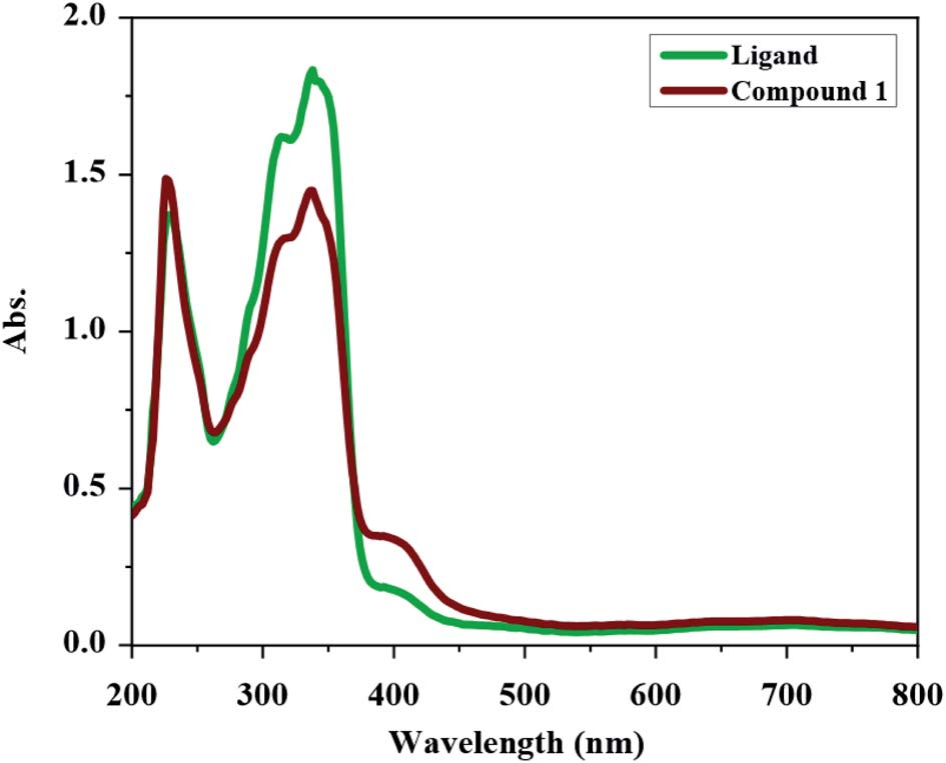
UV-Vis spectrum of H_2_L and compound 1 in methanol (2.5 × 10^−5^ M concentration).

The UV-Vis spectrum of H_2_L and compound 1 were obtained in methanol (see Fig. 1). The UV-Vis spectrum of H_2_L has two absorptions at 338 nm and 228 nm. The band at 338 nm can be devoted to the n → π* transition (characteristic of non-bonded electrons available on C=O, C=N and maybe Ph–NH_2_ and Ph–Br groups) and the higher energy band at 228 nm can be attributed to the π → π* transition (characteristic of the aromatic rings together with C=O and C=N groups). In the UV-Vis spectrum of compound 1, the intensity of the bands is decreased relative to the free ligand which confirm the coordination of ligand to the metal ion. In UV-Vis spectrum of compound 1, the n → π* transition is found at 337 nm and the band at 226 nm is due to the π → π* transition. The new weak broad band at about 400 nm in the UV-Vis spectrum of compound 1 is due to the ligand to the metal charge transfer (LMCT) transition which maybe is the reason of the orange color of this compound.

### X-ray structure of compound 1

**Fig. 2:**
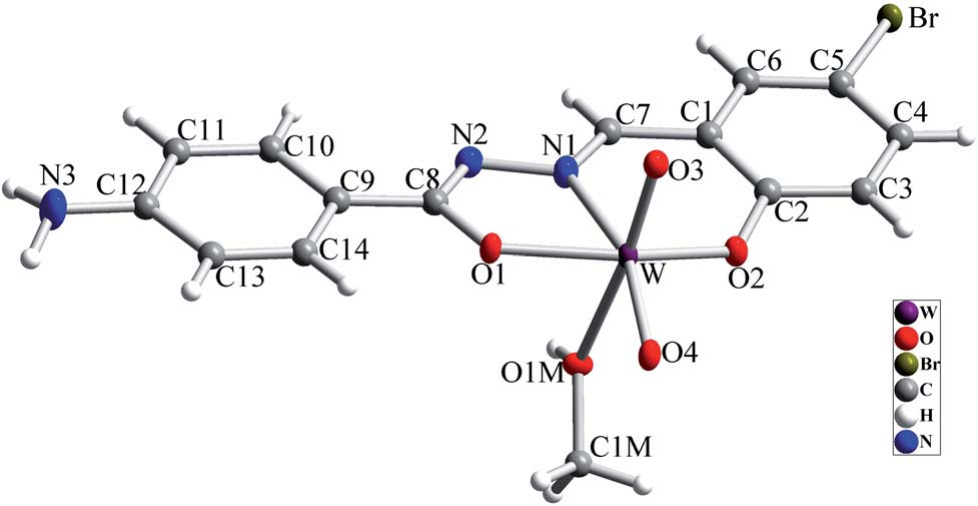
Molecular structure of compound 1 with atom numbering scheme. Ellipsoids are drawn in 30% probability level.

**Fig. 3:**
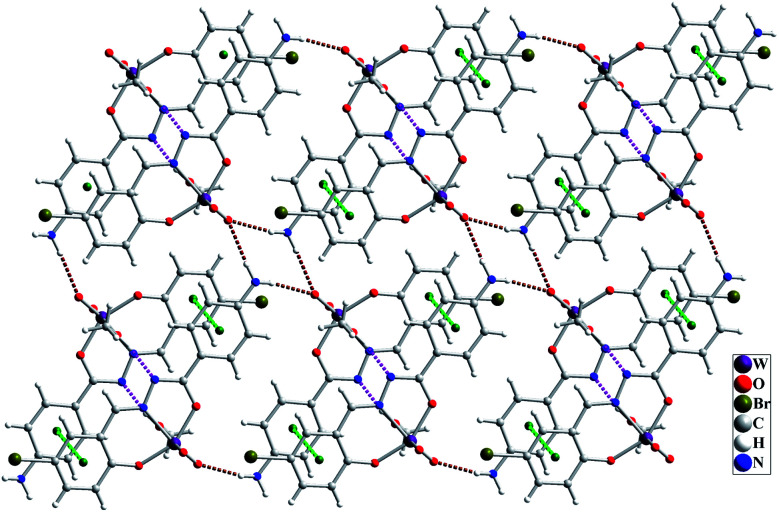
Intermolecular interactions in the crystal structure of compound 1 shown as dashed pink (O–H⋯O), brown (N–H⋯O) and green (π⋯π) dashed lines.

The molecular structure of [WO_2_L(CH_3_OH)] (1) is shown in Fig. 2, and selected bond lengths and bond angles are summarized in Table 1. Single crystal X-ray studies indicated that 1 is a mononuclear W(vi) coordination compound. The tungsten ion in compound 1 is six coordinate and has a distorted octahedral geometry. H_2_L acts as a tridentate O, N, O donor ligand by coordination to the metal ions through its imine nitrogen (N1), amidic oxygen (O1) and phenolic oxygen (O2). The oxygen atom of coordinated methanol (O1M) and two oxygen atoms from two oxido ligands (O3 and O4) fill the remaining sites of the octahedral geometry. Three donor atoms of ligand occupy the three corners of the equatorial plane and the oxygen of one of the oxido groups (O4) occupies the fourth position. In the square plane the W–N1, W–O1, W–O2 and W–O4 distances are 2.242(5), 1.987(5), 1.932(5) and 1.736(5) Å, respectively. The oxygen atoms of methanol molecule (O1M) and remaining oxido group (O3) occupy the axial positions at the distance of W–O1M = 2.313(5) Å and W–O3 = 1.710(6) Å. The W–O1M and W–N1 bonds are located in *trans* position respect to W-oxido groups and have the largest distances from W atom. This matter can be attributed to the high *trans* influence of the oxido groups which elongates these bonds.^22^ By considering the elimination of phenolic and amidic hydrogen atoms during the coordination of ligand to the metal ion, it is obvious that the hydrazone ligand acts as a dianionic ligand (L^2−^) in the iminol form. The C8–N2 and C8–O1 bonds are 1.329(9) and 1.337(8) Å, respectively which are close to the bond lengths of coordinated hydrazone ligands in the iminol form.^23^ The crystal structure of compound 1 confirmed that the NH_2_ group connected to the phenyl ring is remained intact during the formation of Schiff base ligand and it has not reacted with aldehydes and also is not coordinated to the metal ion. By considering the presence of NH_2_ functionality, coordinated methanol group and also the presence of several oxygen and nitrogen atoms in the structure of compound 1, there are intermolecular O–H⋯N and N–H⋯O hydrogen bond interactions in the crystal structure of this compound (see Table 2). Two molecules of the compound 1 connect together by O–H⋯N hydrogen bond interaction and (see Fig. S10†) to form pseudo-dinuclear unit which is also stabilized by strong π⋯π interaction between the phenyl rings. These dinuclear units further connect together by N–H⋯O hydrogen bond interactions to form a polymeric chain (see Fig. 3).

**Table 1:** Selected bond lengths and angles in the crystal structure of compound 1

Bond	Length/Å	Bond	Angle/°
W–O3	1.710(6)	O3–W–O4	105.2(2)
W–O4	1.736(5)	O3–W–O2	97.9(2)
W–O2	1.932(5)	O4–W–O2	101.7(2)
W–O1	1.987(5)	O3–W–O1	96.3(2)
W–N1	2.242(5)	O4–W–O1	98.4(2)
W–O1M	2.313(5)	O2–W–O1	151.3(2)
C2–O2	1.357(8)	O3–W–N1	97.4(2)
C8–O1	1.337(8)	O4–W–N1	156.4(2)
C8–N2	1.329(9)	O2–W–N1	81.9(2)
C12–N3	1.370(9)	O1–W–N1	71.70(19)
C7–N1	1.301(9)	O3–W–O1M	172.08(19)
N1–N2	1.395(8)	O4–W–O1M	82.4(2)
		O2–W–O1M	82.6(2)
		O1–W–O1M	80.0(2)
		N1–W–O1M	74.84(19)

**Table 2:** Hydrogen bonding interactions in the crystal structure of 1^a^

D–H⋯A	D–H/Å	H⋯A/Å	D⋯A/Å	D–H⋯A/°
N3–H31⋯O4^i^	0.91	2.16	3.031(8)	159
N3–H32⋯O4^ii^	0.91	2.37	3.233(8)	158
O1M–H1M⋯N2^iii^	0.84	1.88	2.708(8)	170

a Symmetry codes: (i) −*x* + 2, −*y* + 2, −*z*; (ii) *x* + 1, *y* + 1, *z*; (iii) −*x* + 2, −*y* + 2, −*z* + 1.

### Characterization of the supported catalyst (Si-1)

**Scheme 2:**
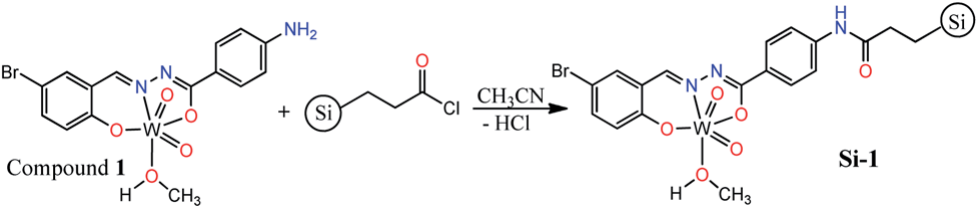
Synthetic pathway of supported catalyst.

By considering the presence of free Ph–NH_2_ functionality in the structure of compound 1, we interested to support it on silica gel and convert into a silica-supported heterogeneous catalyst by the simple amidification reaction (Scheme 2). The supporting process was carried out in dried acetonitrile by the reaction of compound 1 and propionyl chloride-functionalized silica gel (≈1 mmol g^−1^ loading). The silica supported compound 1 (Si-1) was obtained as brown powder after filtration and washing the remained solid by water and methanol. According to the yield of the reaction (91.58%), the loading of compound 1 is approximately 0.65 mmol per one gram of the final solid which is also in agreement with the results of elemental and TGA analyses. Supporting of compound 1 on the surface of silica gel was also confirmed by FT-IR spectroscopy, TGA, DRS, XRD, EDX-map, SEM and XPS analyses. Comparing FT-IR spectrum of Si-1 (Fig. S11†) with starting propionyl chloride-functionalized silica gel (Fig. S12†) confirms the supporting of compound 1 on the surface of the silica. There are three bands at 1096, 812 and 472 cm^−1^ in the FT-IR spectrum of propionyl chloride-functionalized silica gel that are similar to the bands at 1081, 794 and 466 cm^−1^ in the FT-IR spectrum of Si-1. These bands are due to the influence of the absorption of the silica gel.^24^ The band at 3444 cm^−1^ is associated with the coordinated methanol molecule and other OH groups of the support. The band at 1633 cm^−1^ is due to the amidic C=O vibration which confirms the formation of amide functionality from the reaction of compound 1 and propionyl chloride-functionalized silica gel. TGA diagram of the supported catalyst exhibits three steps of weight losses (Fig. S13†). The first step can be attributed to the loss of the water and solvent molecules from the supported catalyst. The second (240 °C to 570 °C) and third steps (830 °C to 950 °C) correspond to the removal of organic ligand and decomposition of the supported coordination compound from the structure of the catalyst. It should be noted that the catalyst is stable up to 380 °C and the main part of organic ligand is removed after this temperature. It is clear that the coordinated methanol molecules are removed in the range of 240–380 °C and the organic ligand is removed after this temperature. The 30.55% of the starting weight is removed up to 950 °C which is in agreement with the supporting of compound 1 on the silica. The remaining weight is related to the silica, tungsten oxide and maybe organic residues that are remained on the surface of silica.

**Fig. 4:**
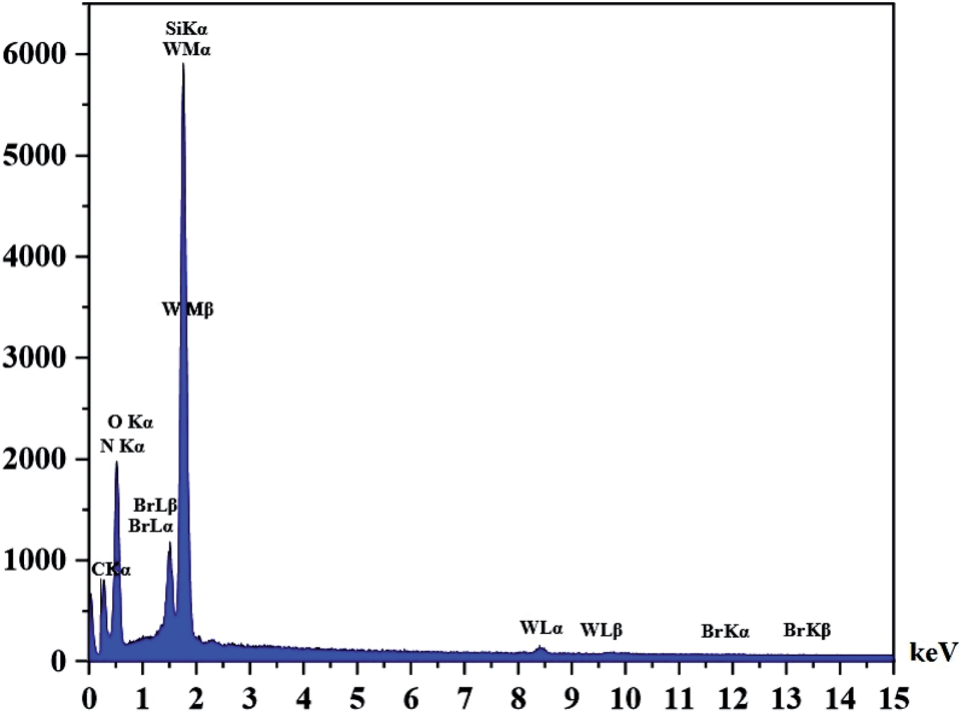
EDX spectrum of the supported catalyst (Si-1).

**Fig. 5:**
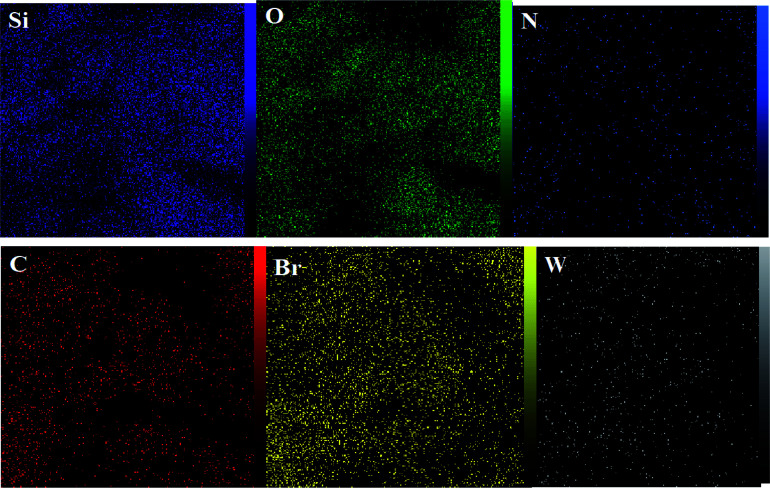
EDX mapping images of the heterogeneous catalyst (Si-1).

**Fig. 6:**
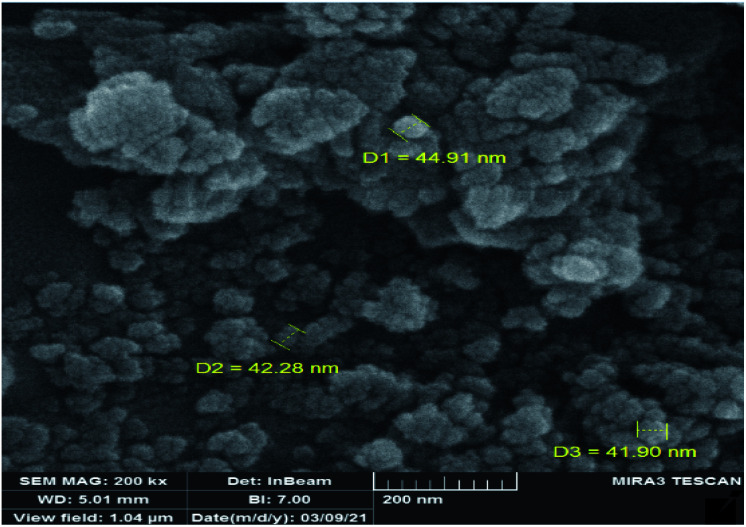
SEM image of the supported catalyst (Si-1).

The DRS spectrum of the heterogeneous catalyst (Fig. S14†) is in consistent with the UV-Vis spectrum of compound 1. In the DRS spectrum of supported catalyst, the peaks at 330 and 360 nm were attributed to n → π* and LMCT transitions, respectively. The EDX analysis of the supported catalyst (see Fig. 4) shows the presence of W atoms as well as Si, O, N, C and Br atoms in the texture of the heterogeneous catalyst. As it is seen, the chloride anion (related to starting propionyl chloride functionality) is absent in the structure of the supported catalyst, which indicates hydrogen chloride is removed during the supporting process. EDX analysis also confirms that compound 1 is successfully supported on the silica by amidification reaction. The EDX mapping images of the supported catalyst is shown in Fig. 5 which indicates the tungsten species have been distributed uniformly in the catalyst texture. In order to study the particle size and morphology of the heterogeneous catalyst, SEM image of the supported catalyst was also provided which is shown in Fig. 6. According to the SEM image, most of the particles are spherical and have diameters in the range of 40–50 nm. The change in the morphology of the catalyst surface is another document for the placement of the tungsten coordination compound on the silica. The compositions of Si-1 and propionyl chloride-functionalized silica gel were investigated by X-ray diffraction analysis (Fig. S15†). The XRD pattern of the pure silica showed high crystalline peaks at 2*θ* = 22.5, 28.3, 40.5, 50.1, 66.2 and 73.8°. According to the XRD pattern of Si-1, the peaks at 2*θ* = 24.7, 30.5, 33.3, 39.2 and 48.2° are in agreement with the XRD pattern of pure silica and the sharp peaks at 2*θ* = 10.9, 12.4, 17.16 and 20.8° are characteristic of the compound 1. The XRD pattern of Si-1 and propionyl chloride-functionalized silica gel indicated that compound 1 is successfully supported on silica gel and heterogeneous catalyst was synthesized.

**Fig. 7:**
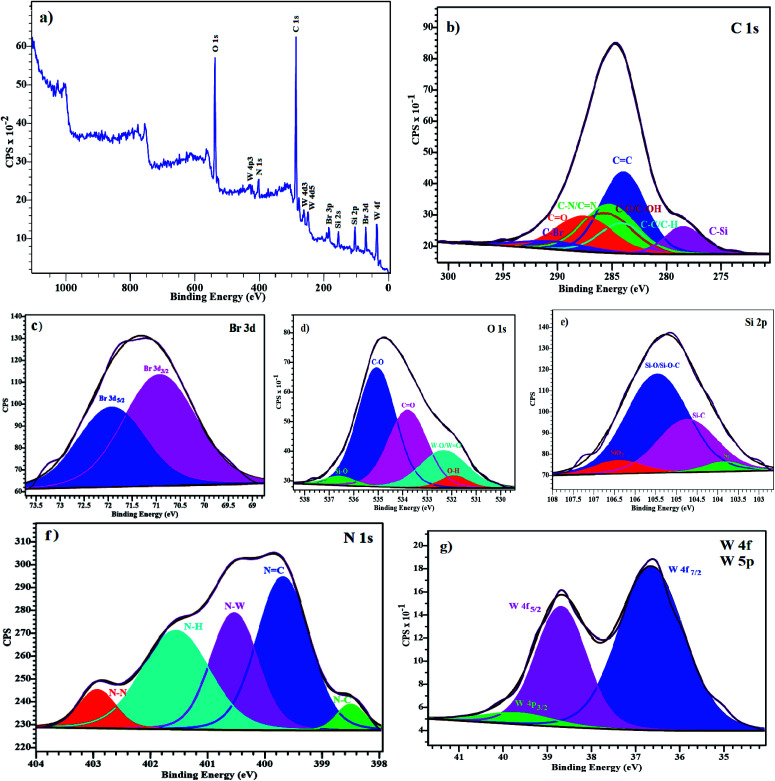
XPS analysis of the supported catalyst (a) survey spectrum (b) C 1s spectrum (c) Br 3d spectrum (d) O 1s spectrum (e) Si 2p spectrum (f) N 1s spectrum (g) W 4f/5p spectrum.

The X-ray photoelectron spectroscopy (XPS) analysis was carried out to investigate the surface composition and oxidation state of the elements on the surface of the supported catalyst. The shifts were corrected by using the standard peak of C 1s as a reference. Results indicate that the elements like W, Si, Br, C, N and O are detected on the surface of the catalyst (Fig. 7a). The high resolution XPS spectra of the C, N, O, Si, Br and W are shown in Fig. 7b–g. The C 1s spectrum can be divided into five peaks. In the C 1s binding energy region, (C–C/C–H), C=C, (C–N/C=N), (C–O/C–OH) and C=O are observed at 285.27, 285.20, 285.71, 285.88 and 286.63 eV, respectively and the peaks at 283.0 and 287.78 eV are related to the C–Si and C–Br (Fig. 7b). As shown in Fig. 7c, the Br 3d spectrum contains two peaks due to spin–orbit (*I* = 3/2, 5/2) coupling. In this region, two peaks are observed at 70.92 and 71.92 eV attributed to the Br 3d_5/2_ and Br 3d_3/2_, respectively. The O 1s spectrum displays two peaks at 535.05 and 533.79 eV which are attributed to C–O and C=O, respectively. A new peak at 532.32 eV is related to the tungsten oxide (W–O/W=O) and two weak peaks at 531.89 and 536.56 eV can be attributed to the O–H and Si–O (Fig. 7d). The Si 2p spectrum, shows two main components corresponding to Si–C and Si–O/Si–O–C bonds at 104.75 and 105.47 eV, respectively and the peaks at 103.86 and 106.41 eV are respectively related to Si and SiO_2_ (Fig. 7e). The N 1s spectrum shows a broad peak that can be divided into three main components that is close to expected of the molecular structure. The main peaks at 399.76, 400.66 and 401.73 eV are assigned to the C=N, W–N and N–H groups, respectively. Two smaller peaks at 398.49 and 403.21 eV can be attributed to the C–N and N–N groups, respectively (Fig. 7f). Significant amounts of W are observed in XPS spectrum, Fig. 7g shows the W 4f and W 4p high resolution XPS spectrum with a double peak of W 4f at 36.65 and 38.69 eV which corresponds to the W 4f_7/2_ and W 4f_5/2_, respectively and the peak corresponding to the W 4p_3/2_ is located at of 39.66 eV.

### Epoxidation of cyclooctene

**Fig. 8:**
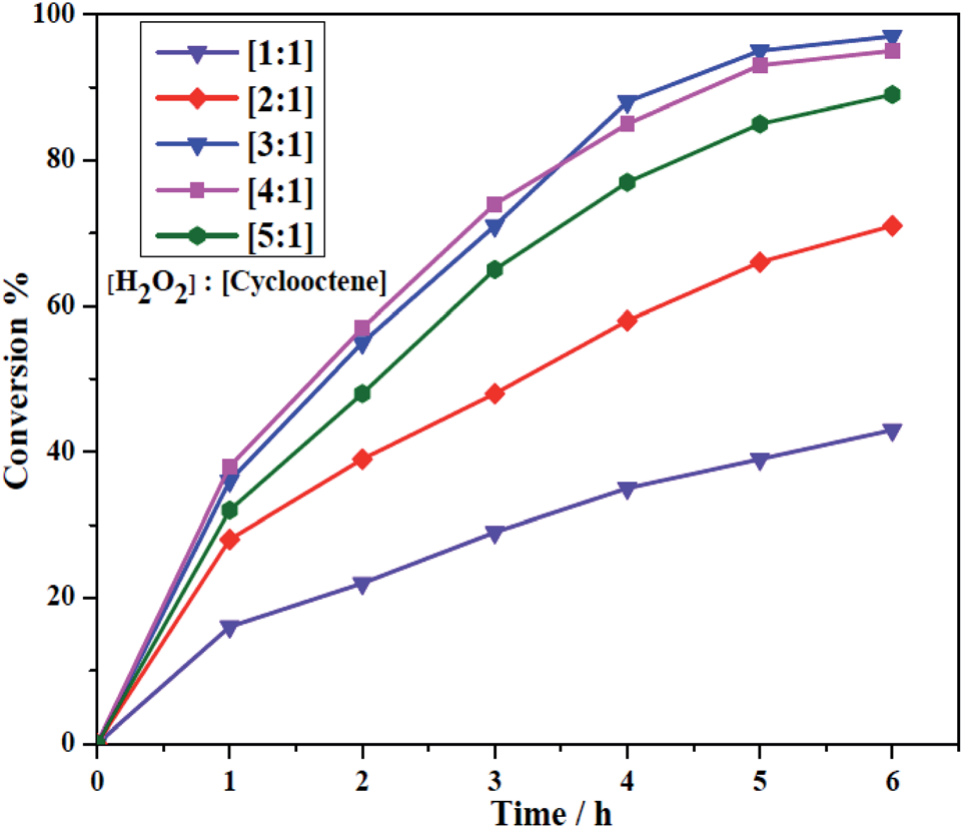
The effect of the concentration of H_2_O_2_ in the catalytic epoxidation of cyclooctene by Si-1.

The obtained heterogeneous catalyst (Si-1) was employed for the epoxidation of olefins and cyclooctene was used as a model substrate in optimization reactions. Aqueous hydrogen peroxide as a green oxidant was used by considering its green character. The reactions were done under solvent free condition by using olefin, H_2_O_2_ and Si-1 at aerial atmosphere. It should be noted that H_2_O_2_ (37% in H_2_O) has considerable amount of water and both of the employed olefins and their epoxidation products are also liquid in the reaction condition. Therefore, removing solvent did not have negative effect on the mass transfer process and interactions between substrate, oxidant and catalyst. Some of the effective parameters, such as the oxidant concentration and temperature of the epoxidation reaction were investigated to achieve the maximum conversion of substrate. Different amounts of the catalyst were also examined in order to obtain the optimal amount of the catalyst. The results of the optimization reactions are collected in Tables 3 and 4. Control experiments indicated there is no product in the absence of catalyst. Also, the epoxidation reaction did not take place even by using higher amount of catalyst in the absence of hydrogen peroxide. This matter showed that the presence of both oxidant and catalyst is urgent for starting epoxidation reaction. Moreover, it can be concluded that the aerial oxygen, as one of the possible oxidants, can not involve in this catalytic system and has no effect on the conversion of substrates to product. For studying the effect of hydrogen peroxide concentration on the epoxidation of cyclooctene, different amounts of oxidant (1, 2, 3, 4 and 5 mmol) were employed in the presence of constant amounts of cyclooctene (1 mmol) and catalyst (5.0 mg, ≈3.25 μmol W). These reactions were also carried out at constant temperature (70 °C). As shown in Fig. 8, the conversion of cyclooctene was firstly increased by increasing the amount of hydrogen peroxide from 1 mmol to 3 mmol but, in the presence of higher amounts of H_2_O_2_ the conversion was almost constant (4 mmol) or slightly decreased (5 mmol). The maximum conversion was obtained when the oxidant to substrate ratio was 3 : 1. Decreasing catalytic activity and low conversion in the presence of higher amount of H_2_O_2_ (5 mmol) maybe is related to the formation of catalytically inactive species or decreasing stability of catalyst in this case. The influence of temperature on the catalytic epoxidation of cyclooctene in the presence of Si-1 was also investigated by performing the reactions at room temperature (≈25 °C), 40, 60, 70, 80 and 90 °C (see Table 3). At room temperature the conversion was very low but the conversion was increased by increasing temperature to 80 °C. Further increasing temperature to 90 °C not only did not improve the conversion, but also decreased the conversion of the reaction. This matter maybe is related to the decomposition of hydrogen peroxide at higher temperatures. Since there was not considerable difference in the conversion of 70 and 80 °C, the former one was selected as the best temperature for this catalytic reaction. Finally, the effect of the amount of catalyst was investigated by using various amounts of catalyst which the results are shown in Table 4. By using 3, 5, 7, 10 and 13 mg of heterogeneous catalyst in the same condition (1 mmol cyclooctene, 3 mmol H_2_O_2_, 70 °C) it was found that the conversion was increased by increasing the amount of catalyst. This matter can be attributed to the increase of the catalytically active centers by increasing the amount of catalyst. Although the increase of the conversion from 3 to 7 mg of catalyst was considerable, there is no high difference between 7 and 10 or 13 mg of catalyst.

**Table 3:** Optimization of the reaction condition in the epoxidation of cyclooctene catalyzed by Si-1 under solvent free condition^a^

Entry	Catalyst (mg)	H_2_O_2_ (mmol)	Temp. (°C)	Conversion (%)
1	—	3	70	0
2	5	—	70	0
3	5	1	70	43
4	5	2	70	71
5	5	3	70	97
6	5	4	70	95
7	5	5	70	89
8	5	3	25	16
9	5	3	40	55
10	5	3	60	85
11	5	3	80	98
12	5	3	90	93

a Reaction condition: cyclooctene (1.0 mmol), reaction time 6 h.

**Table 4:** Effect of the amount of catalyst in the epoxidation of cyclooctene catalyzed by Si-1^a^

Entry	Si-1 (mg)	Conversion^b^ (%)
1	3.0	79
2	5.0	97
3	7.0	99
4	10.0	99
5	13.0	100

a Reaction condition: cyclooctene = 1.0 mmol, H_2_O_2_ = 3 mmol; temperature = 70 °C, reaction time = 6 h.

b Conversions are based on the starting substrate.

In the next step, the catalytic potential of Si-1 was investigated in the epoxidation of some other olefins in the optimized condition (5 mg Si-1, 1 mmol substrate, 3 mmol H_2_O_2_, 70 °C, 6 h). Various alkenes including cyclohexene, styrene, α-methyl styrene, 1-heptene, 1-octene, 1-decene, 2-heptene, 2-octene and *cis*-stilbene were investigated and the results are collected in Table 5. The results indicated that this catalytic system is considerably selective in the epoxidation of cyclooctene, 1-heptene, 1-octene, 1-decene, 2-heptene and 2-octene because only the epoxide product was obtained in these substrates. Nevertheless, in the case of styrene, α-methyl styrene and cyclohexene other byproducts were also observed. Benzaldehyde and acetophenone were detected as byproduct in the oxidation of styrene and α-methyl styrene, respectively. These products were obtained from over-oxidation of the related epoxide products. It should be noted that benzoic acid, as the next byproduct from over-oxidation of benzaldehyde, was not detected in the oxidation of styrene in the presence of Si-1. Styrene is more active than α-methyl styrene which can be attributed higher steric effect of –CH_3_ group in the latter case. Cyclohexen-2-ol and cyclohexen-2-one were detected as byproducts in the oxidation of cyclohexene. Cyclohexene is more active than cyclooctene which is related to its higher potential to involve in allylic oxidation beside the epoxidation of C=C bond. Considering the results of Table 5 indicates both of the electronic and steric effects of the substituents connected to the C=C bond of olefins have considerable effect on the activity and selectivity of this catalytic system. The conversion of cyclic and internal alkenes is higher than terminal alkenes. Since the electron density of C=C bond in internal alkenes is higher than terminal alkenes, they are more favorable for oxidation reaction. On the other hand, comparing conversion of 1-heptene, 1-octene and 1-decene indicates their conversion decreases by increasing the length of carbon chain. This matter can be attributed to the steric effect of the group connected to the C=C bond which is higher in the case of 1-decene. Higher activity of styrene, as the terminal alkene, respect to aliphatic terminal alkenes can be related to the electronic effect of phenyl ring which facilitate the formation of intermediates. Finally, conversion of *cis*-stilbene is considerably lower than other internal alkenes which can be related to high steric hindrance of two phenyl rings in the structure of this substrate. This matter indicates steric effect has higher influence than electronic effect in the epoxidation of alkenes in this catalytic system.

**Table 5:** Catalytic epoxidation of various alkenes with H_2_O_2_ catalyzed by Si-1^a^

Entry	Substrate	Conversion^b^ (%)	Product(s)	Yield (%)	Selectivity (%)
1	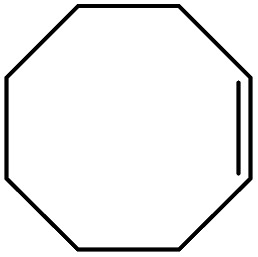	97	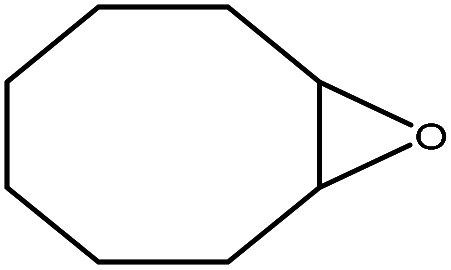	97	100
2	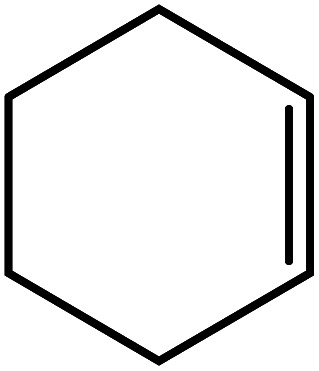	100	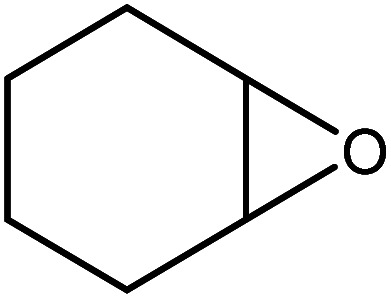	45	45
			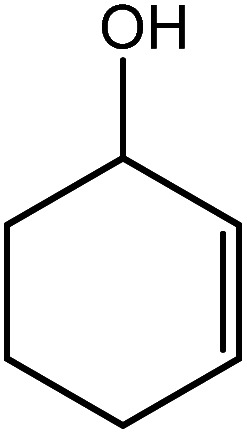	20	20
			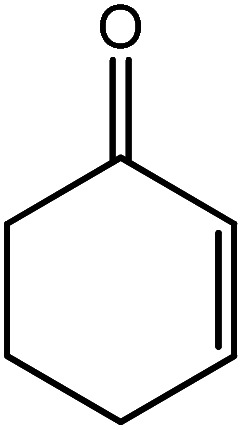	35	35
3	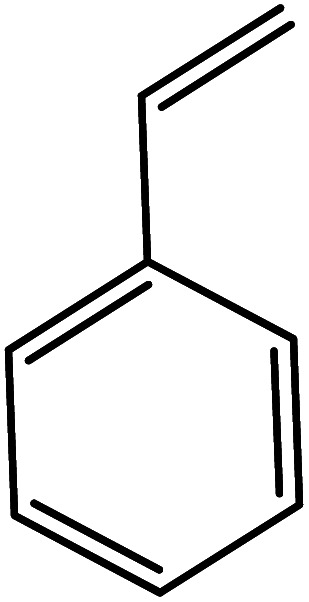	98	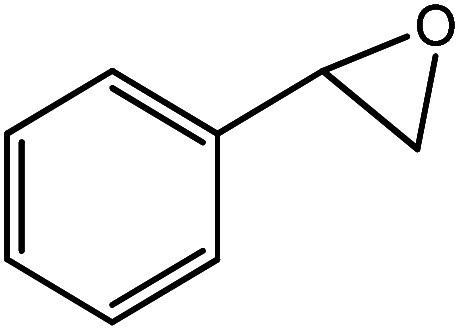	70	71.4
			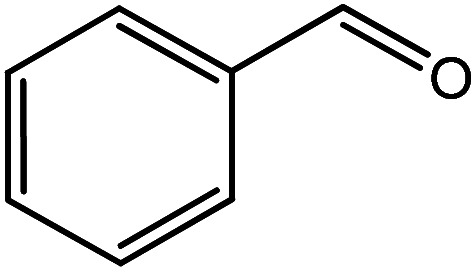	28	28.6
4	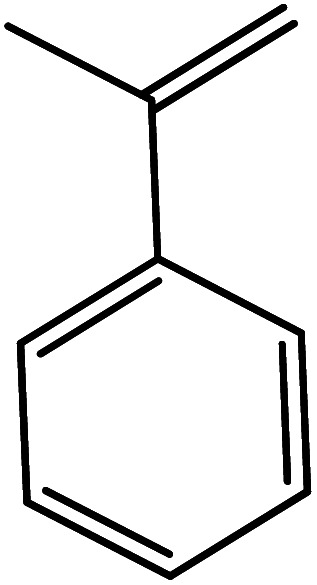	93	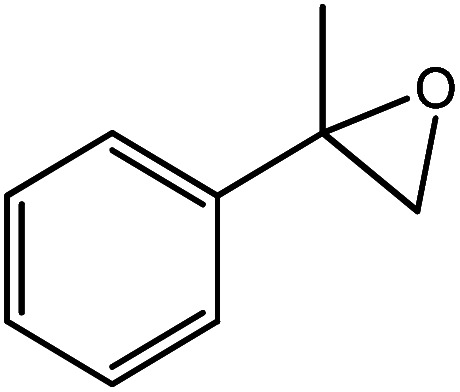	75	80.6
			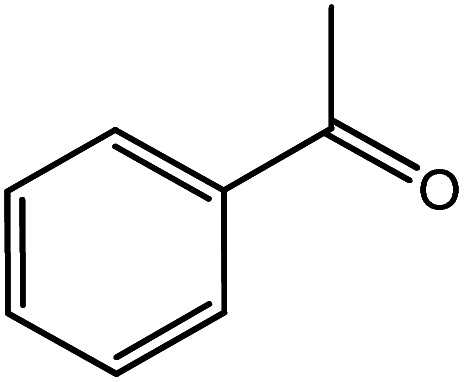	18	19.4
5	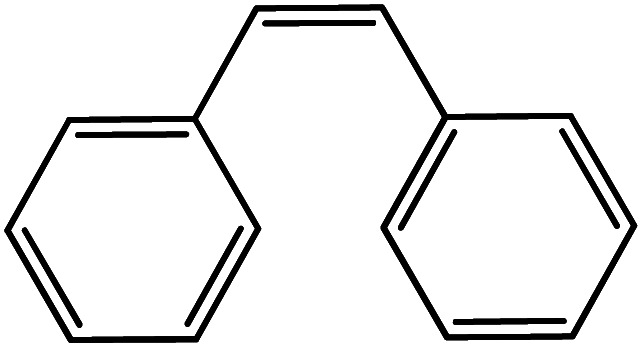	70	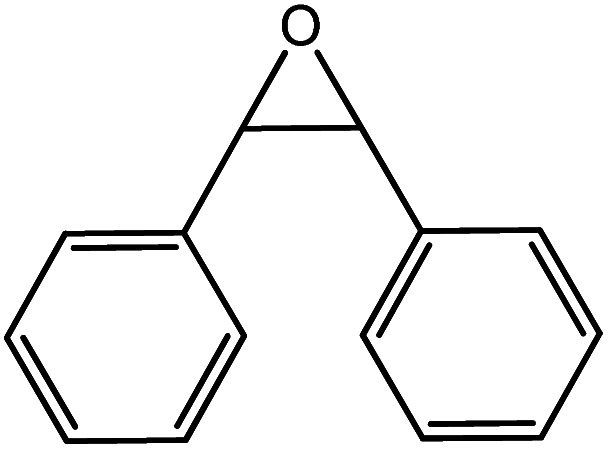	70	100
6	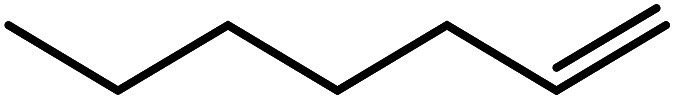	87	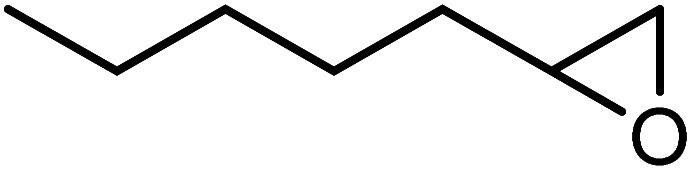	87	100
7	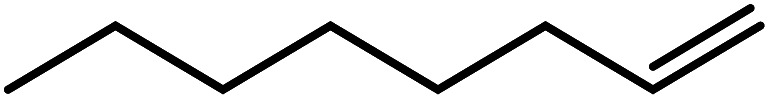	85	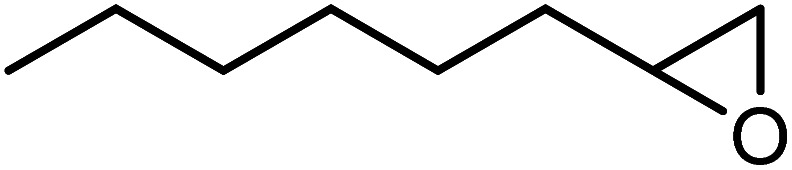	85	100
8	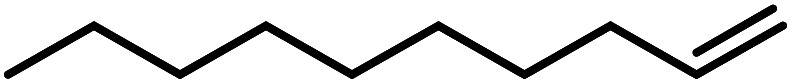	81	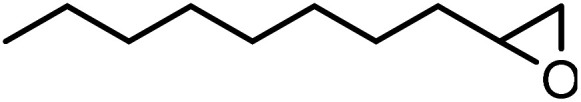	81	100
9	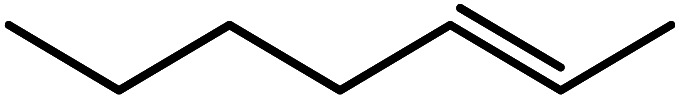	92	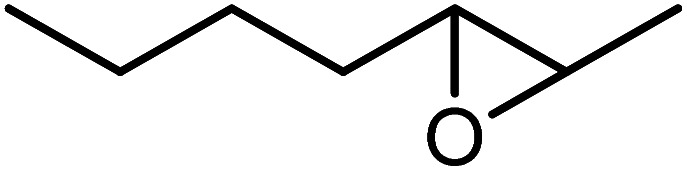	92	100
10	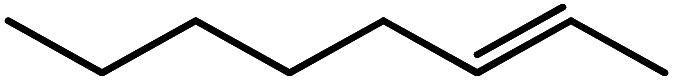	90	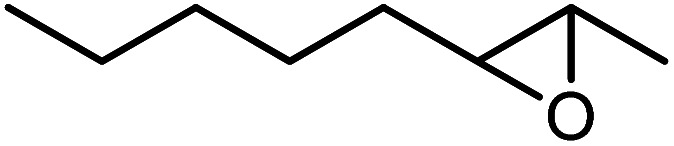	90	100

a Reaction condition: catalyst (5 mg), substrate (1.0 mmol), H_2_O_2_ (3.0 mmol), temperature 70 °C, reaction time 6 h.

b Conversions are based on the starting substrate.

### Characterization of recovered catalyst

The catalyst was recovered by filtration and its catalytic activity was investigated in further epoxidation of cyclooctene in order to check its reusability and stability. The catalyst was successfully recovered several times and the results indicated the activity of the recovered catalyst is comparable with the fresh one. By using 5 mg of catalyst in the optimum condition, 96, 94 and 92% of the cyclooctene was converted to epoxide in the second, third and fourth runs, respectively. Therefore, approximately 5% of the activity of catalyst was decreased after four times recovering process. The recovered catalyst was also characterized by various methods including FT-IR spectroscopy, DRS, TGA, SEM and EDX analyses. In the FT-IR spectrum of the recovered catalyst (see Fig. S16†), the band at 1632 cm^−1^ is assigned to the amidic C=O group. The bands at 1082, 795 and 461 cm^−1^ are attributed to the silica gel and the band at 3446 cm^−1^ is associated with the OH groups. The band at 2925 cm^−1^ is characteristic band of the C–H bonds. The appearance of the characteristic bands of the compound 1 in the FT-IR spectrum of the recovered catalyst indicates that the heterogeneous catalyst is not disturbed even after the epoxidation reaction. TGA analysis of the recovered catalyst (see Fig. S17†) is very similar to the TGA diagram of the fresh catalyst. Just in the first step (in the range of 50–100 °C) relatively higher weight loose is observed which can be attributed to the remove of organic materials and water molecules of oxidant from the recovered catalyst. Similar to TGA diagram of Si-1, there are two steps of the weight losses in the TGA curve of the recovered catalyst. These two main weight losses are due to the removal of the organic moieties. The observed weight loss of 26.02% is in agreement with the structure of the catalyst and confirms the stability of catalyst during catalytic reaction. The DRS spectrum of the recovered catalyst is in consistent with the DRS spectrum of Si-1 and shows broad peaks at 330 and 370 nm which can be attributed due to n → π* and LMCT transitions (Fig. S18†). The slight changes in the shape of spectrum and in the position of peaks are related to the interaction of coordination compound and formation of other oxido–peroxido-tungsten species on the surface of catalyst which is also in agreement with the change in the color of the fresh heterogeneous catalyst (dark brown) compared to the recovered catalyst after the catalytic reaction (light brown).

**Fig. 9:**
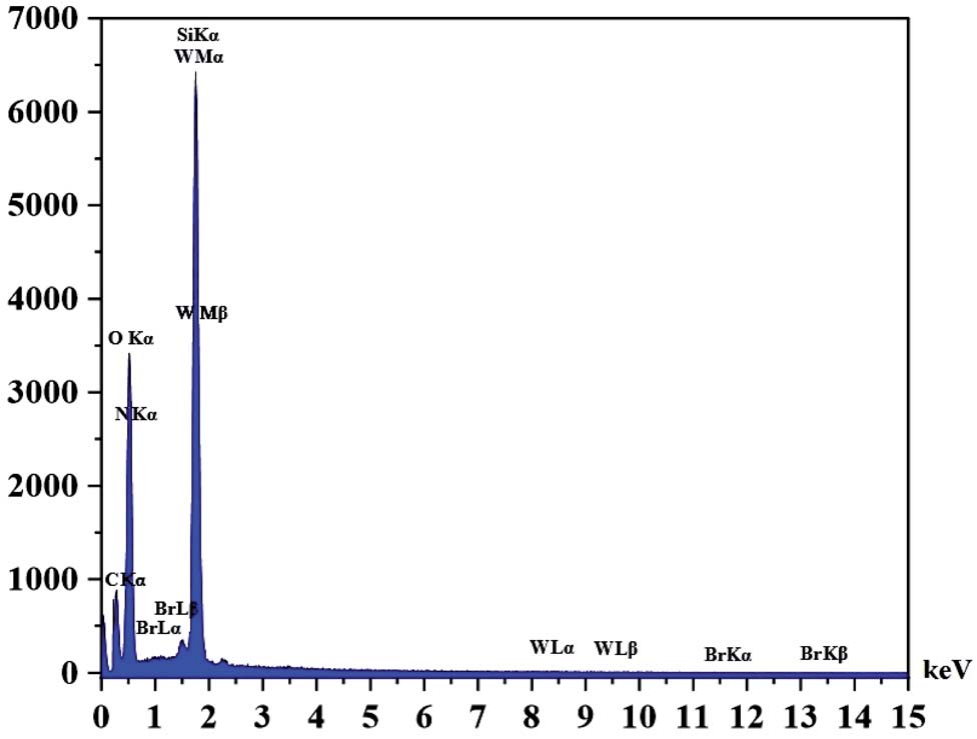
EDX spectrum of recovered catalyst.

**Fig. 10:**
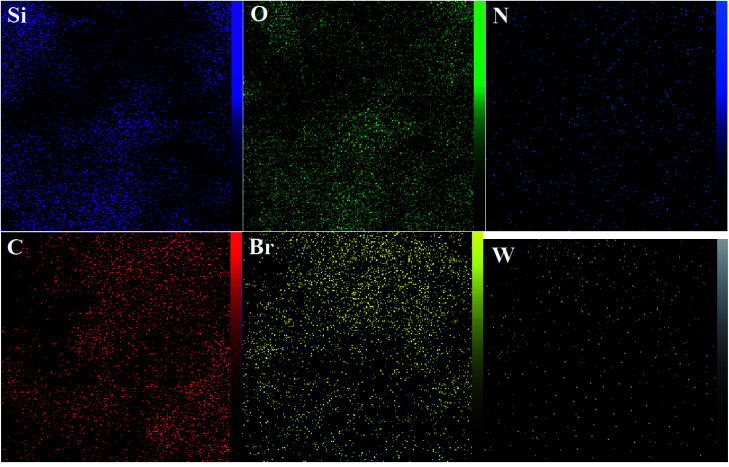
EDX mapping images of the recovered catalyst.

In the EDX analysis of the recovered catalyst the intensity of O has increased in comparison with the fresh catalyst. This matter can be attributed to the effect of hydrogen peroxide and increasing the oxygen content of the catalyst. The presence of W, Si, O, N, C and Br atoms in the EDX analysis of the recovered catalyst confirms the reusability and stability of the catalyst (Fig. 9). The EDX mapping images in the recovered catalyst showed that tungsten species are still uniformly distributed in the catalyst texture (see Fig. 10).

**Fig. 11:**
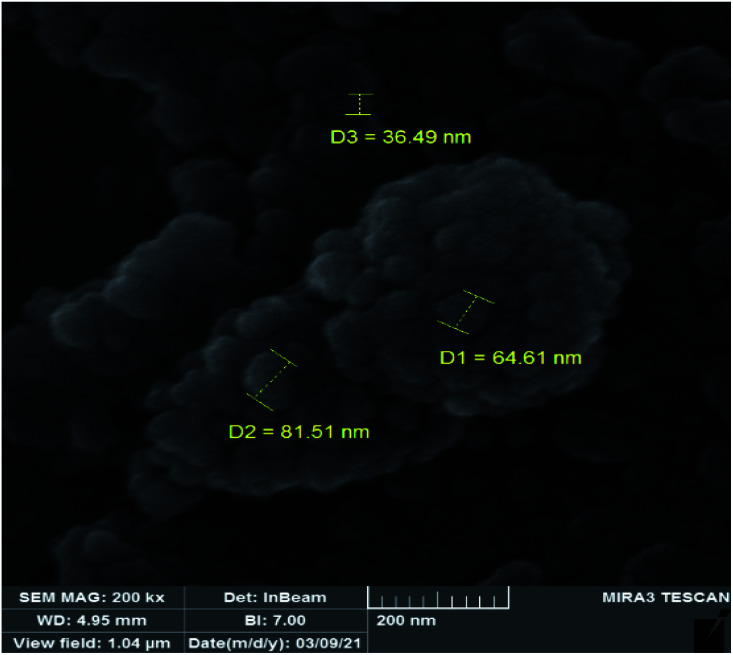
SEM image of the recovered catalyst.

In order to study the changes of the particle sizes and also the morphology of the catalyst after epoxidation reaction, SEM image was also provided for the recovered catalyst. The SEM image of the recovered catalyst is shown in Fig. 11 which indicates it is similar to the fresh catalyst. The shape of particles is spherical but the size of particles is slightly increased to 50–80 nm. Increasing the size of particles can be attributed to sticking of the particles during the catalytic reaction and separation processes. Due to the lack of noticeable changes in the morphology and particle shape of the recovered catalyst, it can be concluded that the heterogeneous catalyst is not degraded in the epoxidation reactions.

### Mechanism of the catalytic epoxidation in the presence of Si-1

**Scheme 3:**
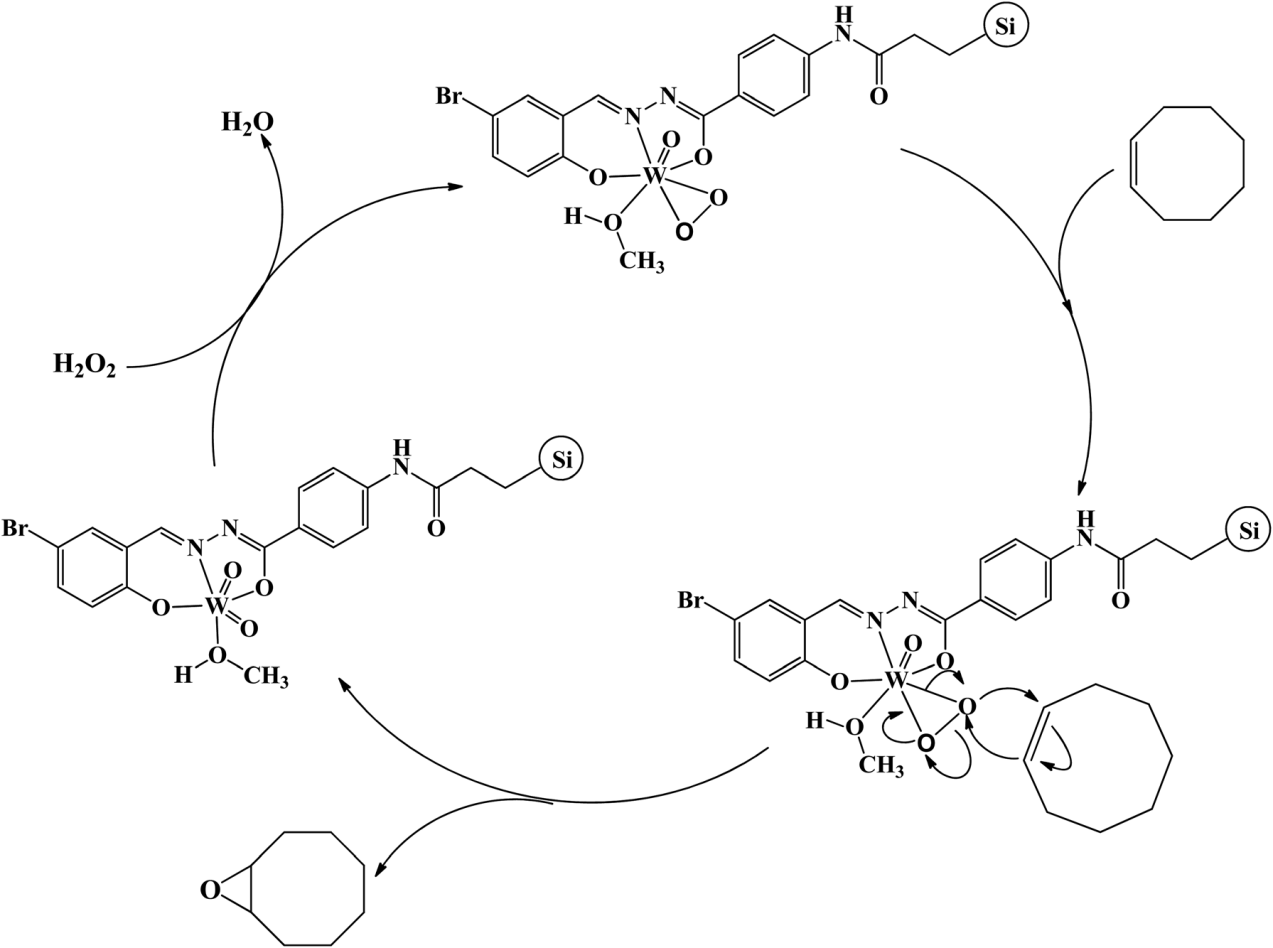
Proposed catalytic mechanism of cyclooctene epoxidation in the presence of Si-1.

Detecting and characterization of catalytically active intermediates in heterogeneous systems have considerable limitations and is a difficult matter. Therefore, in order to find some evidences about the interaction of hydrogen peroxide with compound 1 we interested to check their interaction in the homogeneous condition. For this purpose, diluted hydrogen peroxide (0.1 mmol) in methanol was dropwise added to the methanolic solution of compound 1 (2.5 × 10^−5^ M) and the UV-Vis spectrum of the solution was recorded after successful addition of each drop of diluted hydrogen peroxide. The results of UV-Vis spectroscopy (see Fig. S19†) indicated that the solution of compound 1 is sensitive toward hydrogen peroxide. The shifts of the bands and also the change in their intensity after addition of hydrogen peroxide are mainly related to the formation of peroxido-tungsten species in the solution.^25^ Such species can act as catalytically active intermediates and transfer oxygen atom to the olefins. The role of peroxido-tungsten species as the catalytically active intermediates can be also confirmed by the fact that the epoxidation reaction did not take place in the absence of hydrogen peroxide even in the presence of higher amount of catalyst (0.5 g). These species can also form in the solid state by the interaction of heterogenised compound 1 with hydrogen peroxide. It should be noted that the slight change in the color of heterogeneous catalyst after addition of hydrogen peroxide and catalytic reaction is also related to the formation of such species. By considering these changes and also reported mechanisms for the epoxidation reactions in the presence of tungsten(vi) coordination compounds,^25^ a mechanism was proposed for this catalytic system (see Scheme 3). Similar to the reports in literature,^26^ it is predictable that the oxido–peroxido-tungsten species formed by the interaction of hydrogen peroxide with compound 1 on the surface of the hydrogenised catalyst are active and responsible intermediates for epoxidation of olefins.

## Conclusions

In summary, a new tungsten compound, [WO_2_L(CH_3_OH)], was synthesized and characterized by UV-Vis, FT-IR and NMR spectroscopic methods and also by TGA and single crystal X-ray analyses. Then, compound was supported on the silica gel and a novel silica supported heterogeneous catalyst was synthesized. The heterogeneous catalyst was characterized by FT-IR, DRS, TGA, XRD, EDX, SEM and XPS analyses. The heterogeneous catalyst was investigated in the green epoxidation of olefins in the presence of H_2_O_2_ as oxidant under solvent free condition. The effect of some influencing parameters like concentration of oxidant, the amount of catalyst and temperature were investigated. The results indicated this heterogeneous catalytic system is an active, selective and stable catalyst for green epoxidation of olefins and reaction condition together with electronic and steric properties of the substrate can influence on its activity and selectivity. The catalyst was recovered and characterized by various methods which indicated it is stable after catalytic reaction and can be used for several times without significant loose in its activity.

## Experimental

### Materials and instrumentation

4-Aminobenzoic hydrazide, 5-bromo-2-hydroxybenzaldehyde, tungsten(vi) chloride (WCl_6_), propionyl chloride-functionalized silica gel (1 mmol g^−1^ loading) and solvents were purchased from Sigma-Aldrich and used as received. FT-IR spectra were recorded as KBr disks with a Bruker FT-IR spectrophotometer. The UV-Vis spectra of solution were recorded on a thermos-spectronic Helios Alpha spectrophotometer between 200-800 nm. The ^1^H and ^13^C NMR spectra were taken from Bruker DRX-300 spectrometer in DMSO-d_6_ solution. Diffuse-reflectance UV-Vis spectroscopy (DRS) were recorded with Sinco S4100 instrument. Elemental analyses (C, H, N) were performed on a Carlo ERBA Model EA 1108 analyzer and the tungsten content of the compounds was measured by atomic absorption spectroscopy using a Varian AA-220 instrument. Thermal gravimetric analysis (TGA) curves were taken from TA Q600 instrument in the range of 25–1000 °C. X-ray diffraction analyses (XRD) for heterogeneous catalyst were obtained from PHILIPS PW1730 instrument. Energy-dispersive X-ray spectroscopy (EDX) and scanning electron microscopy (SEM) were recorded with TESCAN MIRA III instrument. X-ray photoelectron spectroscopy (XPS) analysis was performed by using a Bes Tec 8025 instrument.

### Synthesis of (*E*)-4-amino-*N*′-(5-bromo-2-hydroxybenzylidene)benzohydrazide (H_2_L)

4-Aminobenzoic hydrazide (1.00 g, 6.615 mmol) was refluxed with 5-bromo-2-hydroxybenzaldehyde (1.33 g, 6.615 mmol) in methanol (20 mL) for 6 h at 60 °C (Scheme 1a). A large amount of white precipitates was appeared after cooling the reaction flask to room temperature and decreasing the volume of solvent to 5 mL. The resultant precipitate was collected by filtration, washed with cold methanol and dried at room temperature. H_2_L was obtained as pure colorless crystalline material by recrystallization in methanol. Yield: 93.6% (2.07 g). M.p. 258–261 °C. Anal. calc. For C_14_H_12_BrN_3_O_2_ (334.17 g mol^−1^): C, 50.32; H, 3.62; N, 12.57%. Found: C, 50.37; H, 3.59; N, 12.62%. FT-IR (KBr, cm^−1^): 3471 (s), 3354 (s), 3214 (s), 1625 (*vs.*), 1603 (*vs.*), 1570 (m), 1539 (s), 1512 (s), 1475 (s), 1444 (w), 1341 (*vs.*), 1318 (m), 1277 (*vs.*), 1204 (w), 1185 (s), 1152 (m), 1127 (m), 1086 (w), 960 (s), 924 (s), 884 (s), 838 (*vs.*), 815 (s), 780 (w), 762 (s), 748 (w), 704 (w), 691 (s), 657 (s), 627 (*vs.*), 616 (m), 562 (s), 528 (s), 498 (s), 480 (m), 473 (s). ^1^H NMR (300 MHz, DMSO-d_6_, 25 °C, TMS, ppm): *δ* = 5.85 (2H, s, –NH_2_), 6.60 (2H, d, *J* = 8.70 Hz), 6.87 (1H, d, *J* = 8.70 Hz), 7.36 (1H, dd, *J* = 8.70 Hz, ^4^*J* = 2.10 Hz), 7.69 (2H, d, *J* = 7.20 Hz), 7.71 (1H, s), 8.52 (1H, s, –CH=N–), 11.55 (1H, s, –OH), 11.86 (1H, s, –NH–N). ^13^C NMR (75 MHz, DMSO-d_6_, 25 °C, ppm): *δ* = 110.8, 113.1, 119.1, 121.8, 122.2, 130.0, 131.2, 133.5, 145.0, 153.1, 156.8 and 163.3 ppm. UV-Vis (2.5 × 10^−5^ M, CH_3_OH): *λ*_max_ (*ε*, M^−1^ cm^−1^): 228 (52 800), 338 nm (71 350).

### Synthesis of [WO_2_L(CH_3_OH)] (1)

Compound 1 was synthesized in the reaction of H_2_L (0.100 g, 0.299 mmol) and WCl_6_ (0.119 g, 0.300 mmol) in 10 mL methanol by using a branched tube. In this reaction, mentioned amounts of the materials were added to the main arm of branched tube and the tube was filled with methanol. The main arm was placed into an oil bath at 60 °C and the branch was kept at room temperature.^27^ The reaction was started slowly and the color of solution was slowly changed to orange. The orange crystals of compound 1 were directly obtained in the cold arm of the branched tube after about one week. The crystals were collected, washed with cold methanol and dried at air. Isolated yield based on the starting ligand: 60.54% (0.105 g). Anal. calc. For C_15_H_14_BrN_3_O_5_W (580.05 g mol^−1^): C, 31.06; H, 2.43; N, 7.24; W, 31.69%. Found: C, 31.10; H, 2.45; N, 7.20; W, 31.74%. FT-IR (KBr, cm^−1^): 3548 (br), 3440 (br), 3347 (s), 3244 (m), 1627 (s), 1605 (*vs.*), 1548 (s), 1492 (*vs.*), 1470 (m), 1448 (s), 1410 (s), 1372 (*vs.*), 1344 (*vs.*), 1264 (s), 1209 (m), 1181 (*vs.*), 1050 (m), 947 (*vs.*), 938 (s), 914 (m), 902 (s), 834 (s), 811 (m), 669 (s), 636 (w), 429 (m), 419 (m). ^1^H NMR (300 MHz, DMSO-d_6_, 25 °C, TMS, ppm): *δ* = 3.14 (3H, s, –CH_3_), 3.39 (1H, s, OH), 6.01 (2H, s, –NH_2_), 6.59 (2H, d, *J* = 7.25 Hz), 6.94 (1H, d, *J* = 8.75 Hz), 7.63 (1H, d, *J* = 9.00 Hz), 7.69 (2H, d, *J* = 7.25 Hz), 7.91 (1H, s), 8.70 ppm (1H, s, –CH=N–). ^13^C NMR (75 MHz, DMSO-d_6_, 25 °C, ppm): *δ* = 49.1, 113.0, 113.7, 115.4, 122.0, 123.5, 130.7, 135.8, 137.1, 153.6, 157.4, 158.1 and 170.0 ppm. UV-Vis (2.5 × 10^−5^ M, CH_3_OH): *λ*_max_ (*ε*, M^−1^ cm^−1^): 226 (57 350), 337 nm (55 800).

### Effect of water in the formation of [WO_2_L(CH_3_OH)] (1)

By considering the structure of compound 1 and the presence of [WO_2_]^2+^ unit in the structure of product, the reaction of H_2_L and WCl_6_ was performed in 10 mL of dried methanol. Compound 1 did not form in this condition after one week. The reaction was repeated in a mixture of methanol : water solvent. The methanol solvent containing 5, 10 and 20 v/v% of water was firstly prepared by the addition of 0.5, 1.0 and 2.0 mL of water to 9.5, 9.0 and 8.0 mL of methanol, respectively. These solvents were used to fill the branched tube and the results indicated that the rate of reaction was increased by the addition of water to the reaction mixture since the crystals of compound 1 was produced in a shorter time (3 days). This matter confirms the rate of the reaction depends on the amount of water. It should be noted that in the presence of solvent containing 5% of water (9.5 mL methanol + 0.5 mL water) the quality of crystals is better than 10 and 20%. Compound 1 was also formed in the presence of methanol–water solvent under nitrogen atmosphere which confirmed molecular oxygen does not involve in the reaction.

### Synthesis of supported catalyst (Si-1)

Compound 1 was supported on silica by the reaction of propionyl chloride-functionalized silica gel (0.3 g, ≈1 mmol g^−1^ loading) and compound 1 (0.174 g, 0.30 mmol) in 5 mL dried acetonitrile (Scheme 2). Mentioned amounts of materials were added to a flask and the mixture was stirred at room temperature for 6 hours. Then, the mixture was stirred at 80 °C for other 18 hours and the obtained brown solid was separated by filtration and washed with distilled water and then by methanol. Yield based on compound 1: 91.58% (0.424 g). FT-IR (KBr, cm^−1^): 3444 (br), 3358 (br), 3225 (br), 2924 (m), 2853 (w), 1713 (w), 1704 (w), 1633 (s), 1608 (s), 1549 (w), 1497 (w), 1471 (w), 1443 (w), 1399 (m), 1384 (*vs.*), 1341 (w), 1265 (w), 1178 (w), 1081 (br), 956 (s), 817 (br), 794 (br), 742 (w), 548 (w), 524 (w), 466 (*vs.*), 428 (m), 416 (s). Elemental analysis, found: C, 13.30; H, 1.09; N, 2.69; W, 11.81%.

### Catalytic epoxidation reactions

Catalytic epoxidation reactions were carried out by refluxing a mixture containing suitable amount of heterogeneous catalyst, hydrogen peroxide and 1 mmol of olefin under solvent-free condition. Aqueous hydrogen peroxide (1–5 mmol, 30 wt% in H_2_O) was added to the mixture when the flask reached to the desired temperature and the mixture was cautiously stirred. The progress of the catalytic reactions was monitored by using an Agilent 7890B gas chromatography instrument equipped with a HP-5 capillary column (phenyl methyl siloxane 30 m × 320 μm × 0.25 μm). At appropriate times (every one hour) the sample was taken from the reaction mixture and injected into the GC instrument and the conversion of substrate to product was calculated. At the end of the reaction, 5 mL of cold acetonitrile was added to the flask and the heterogeneous catalyst was isolated from the reaction mixture by simple filtration (or decantation), washed three times with cold acetonitrile and after drying, it was used in other catalytic experiments.

### X-ray crystallography

The crystallographic data and structure refinement parameters are given in Table 6. An orange crystal of compound 1 having approximate dimensions 0.29 × 0.23 × 0.21 was selected and intensity data were measured at 150 K. The data were collected using a Kuma KM-4 diffractometer equipped with graphite-monochromated Mo Kα radiation (*λ* = 0.71073 Å) and Sapphire CCD detector. The data collections, unit cell refinements, data reduction and absorption corrections were performed with the CrysAlisPro software package.^28^ The intensity data were collected by *ω*-scan mode within 2.9° < *θ* < 26° for *hkl* (−8 ≤ *h* ≤ 9, −12 ≤ *k* ≤ 12, −13 ≤ *l* ≤ 13) in the triclinic system. The structure was solved by direct methods and refined by full-matrix least-squares based on *F*^2^ by SHELX-2014 program.^29^

**Table 6:** Crystal data and structure refinement parameters for compound 1

Formula	C_15_H_14_BrN_3_O_5_W
*M*_r_/g mol^−1^	580.05
Crystal shape, color	Block, orange
Crystal size/mm	0.29 × 0.23 × 0.21
*T*/K	150
*λ*/Å	0.71073
Crystal system	Triclinic
Space group	P1̄
*a*/Å	7.663(2)
*b*/Å	10.481(3)
*c*/Å	10.845(3)
*α*/°	95.43(3)
*β*/°	93.93(3)
*γ*/°	104.74(4)
*V*/Å^−3^	834.7(4)
*Z*	2
*D*_calc_/g cm^−3^	2.308
*μ*/mm^−1^	9.35
*T*_min_, *T*_max_	0.152, 0.264
*F*(0 0 0)	548
*θ* range/°	2.9–25
*hkl*	−8 → 9 −12 → 12 −13 → 13
*R*_int_	0.045
*R*[*F*^2^ > 2*σ*(*F*^2^)]	0.044
w*R*(*F*^2^)	0.124
*S*	1.09
Abs. correction	Analytical
Hydrogen refinement	Constr
Measured reflections	7989
Independent reflections	3275
Reflections with *I* > 2*σ*(*I*)	3105
Parameters	229
Restraints	0
Δ*ρ*_max_/Δ*ρ*_min_/e Å^−3^	1.36/−1.72

## Conflicts of interest

The authors declare there is no conflicts of interest.

## Supplementary Material

RA-012-D1RA09217K-s001

RA-012-D1RA09217K-s002
