# Supplementary material for: Green solvent free epoxidation of olefins by a heterogenised hydrazone-dioxidotungsten(vi) coordination compound
Source: RSC Adv. 2022 Feb 9;12(8):4813–27. doi: 10.1039/d1ra09217k (PMC8981271; doi:10.1039/d1ra09217k)
Supplement: RA-012-D1RA09217K-s001 [file RA-012-D1RA09217K-s001.pdf]

Electronic Supplementary Material (ESI) for RSC Advances.
This journal is © The Royal Society of Chemistry 2022

Supporting information file

Green solvent free epoxidation of olefins by heterogenised hydrazone-dioxidotungsten(VI) coordination compound

Neda Heydari,[a] Rahman Bikas,[b,*] Maryam Shaterian,[a] Tadeusz Lis[c]

[a] *Department of Chemistry, Faculty of Science, University of Zanjan, 45371-38791, Zanjan, Iran*

[b] *Department of Chemistry, Faculty of Science, Imam Khomeini International University, 34148-96818, Qazvin, Iran. Email addresses: bikas@sci.ikiu.ac.ir; bikas_r@yahoo.com*

[c] *Faculty of Chemistry, University of Wroclaw, Joliot-Curie 14, Wroclaw 50-383, Poland*

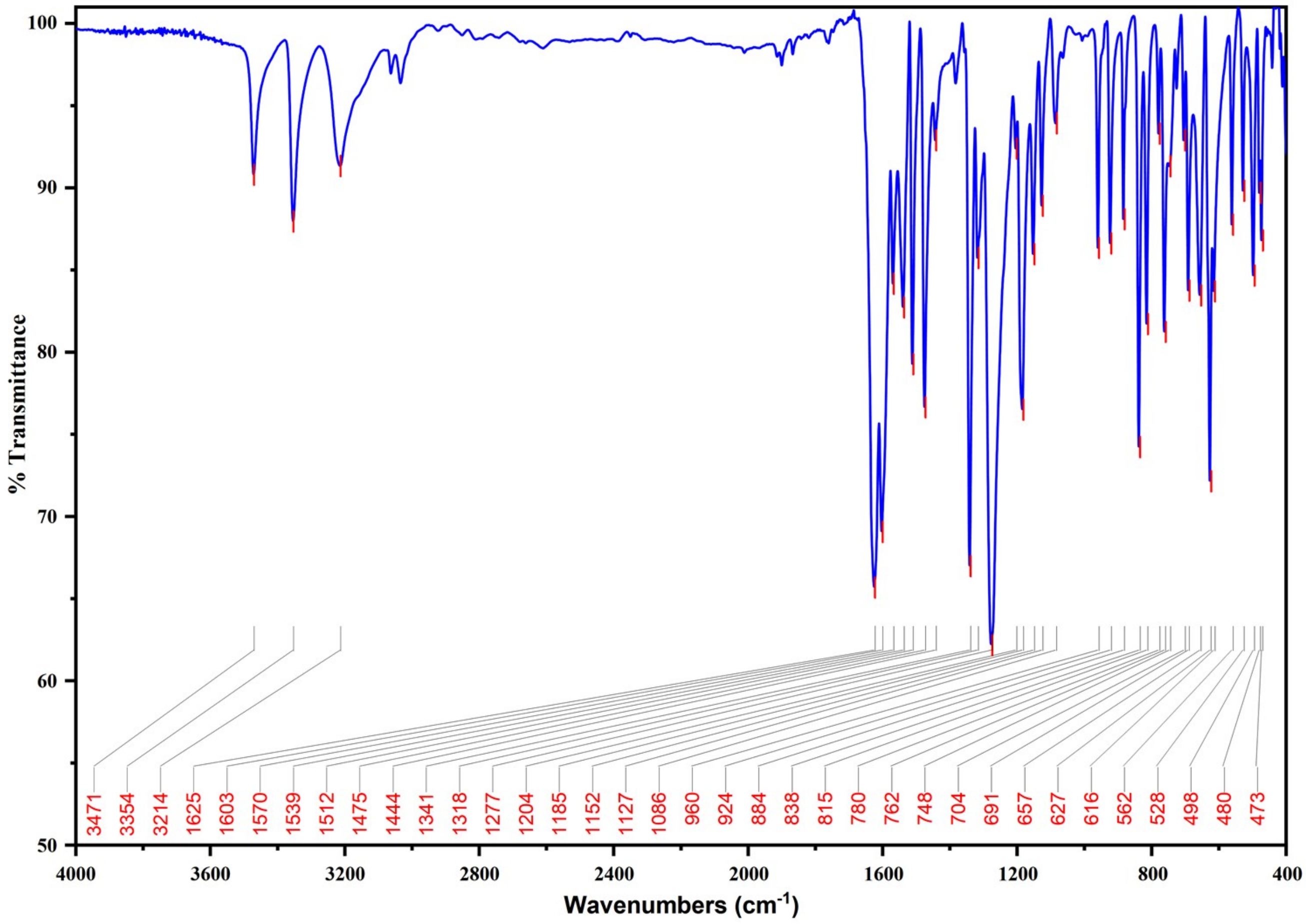

Fig. S1. FT-IR spectrum of H_2L

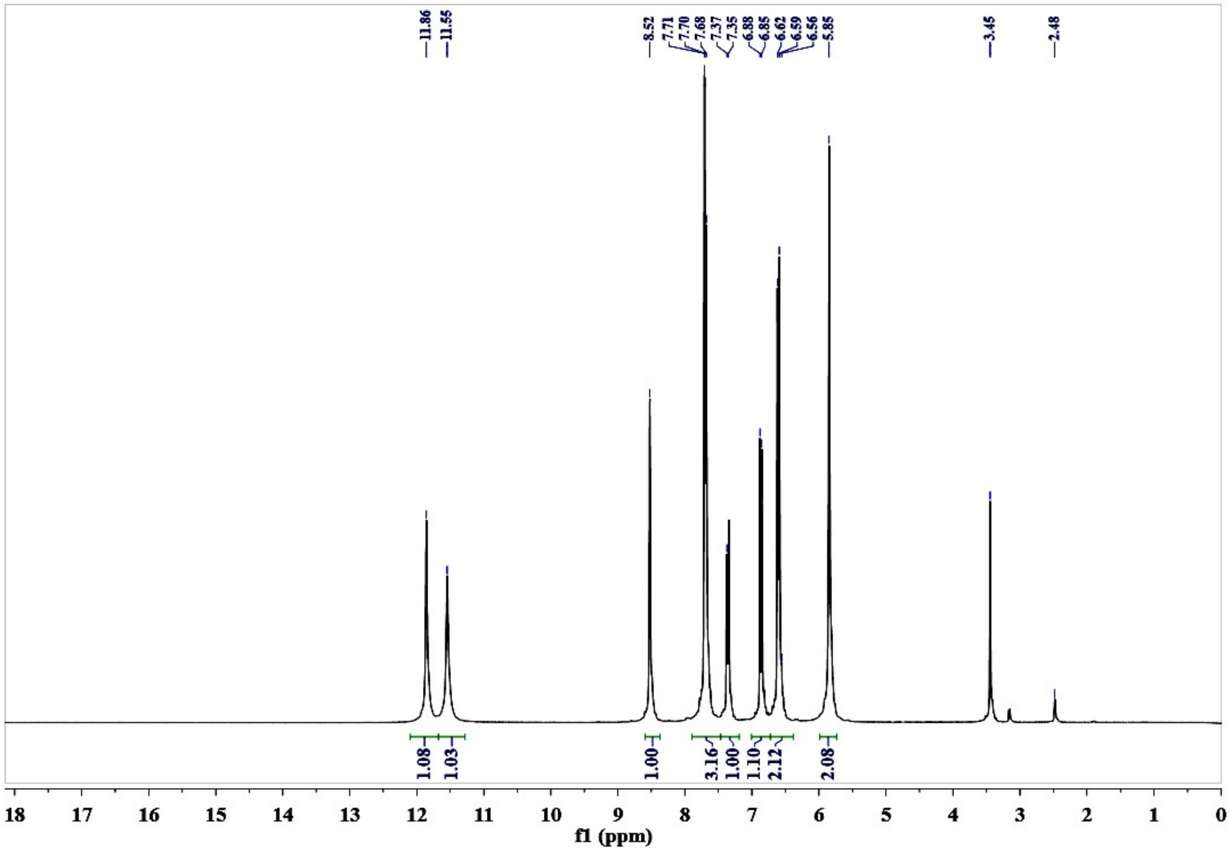

Fig. S2. ^{1}H-NMR spectrum of H_2L in DMSO-d_6

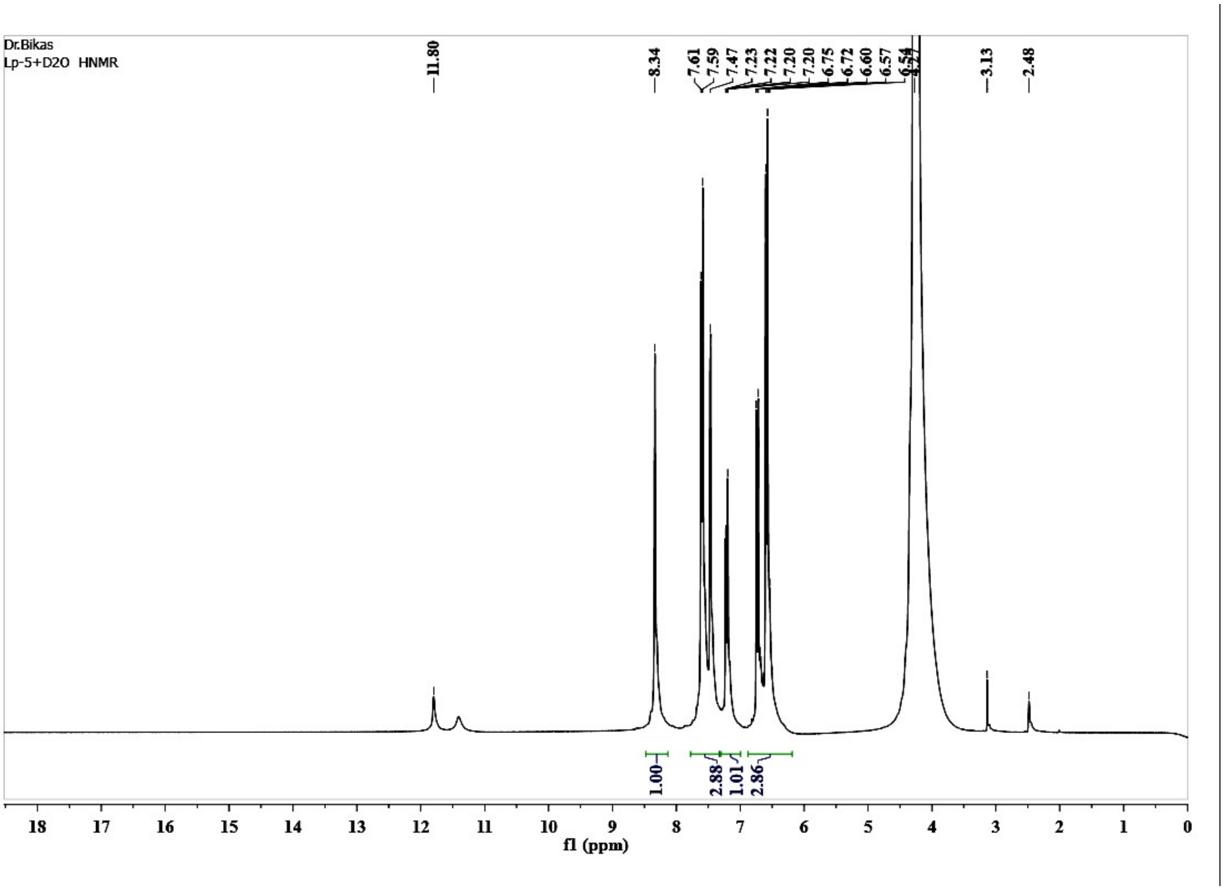

Fig. S3. ^{1}H-NMR spectra of H_2L in DMSO-d_6 + D_2O

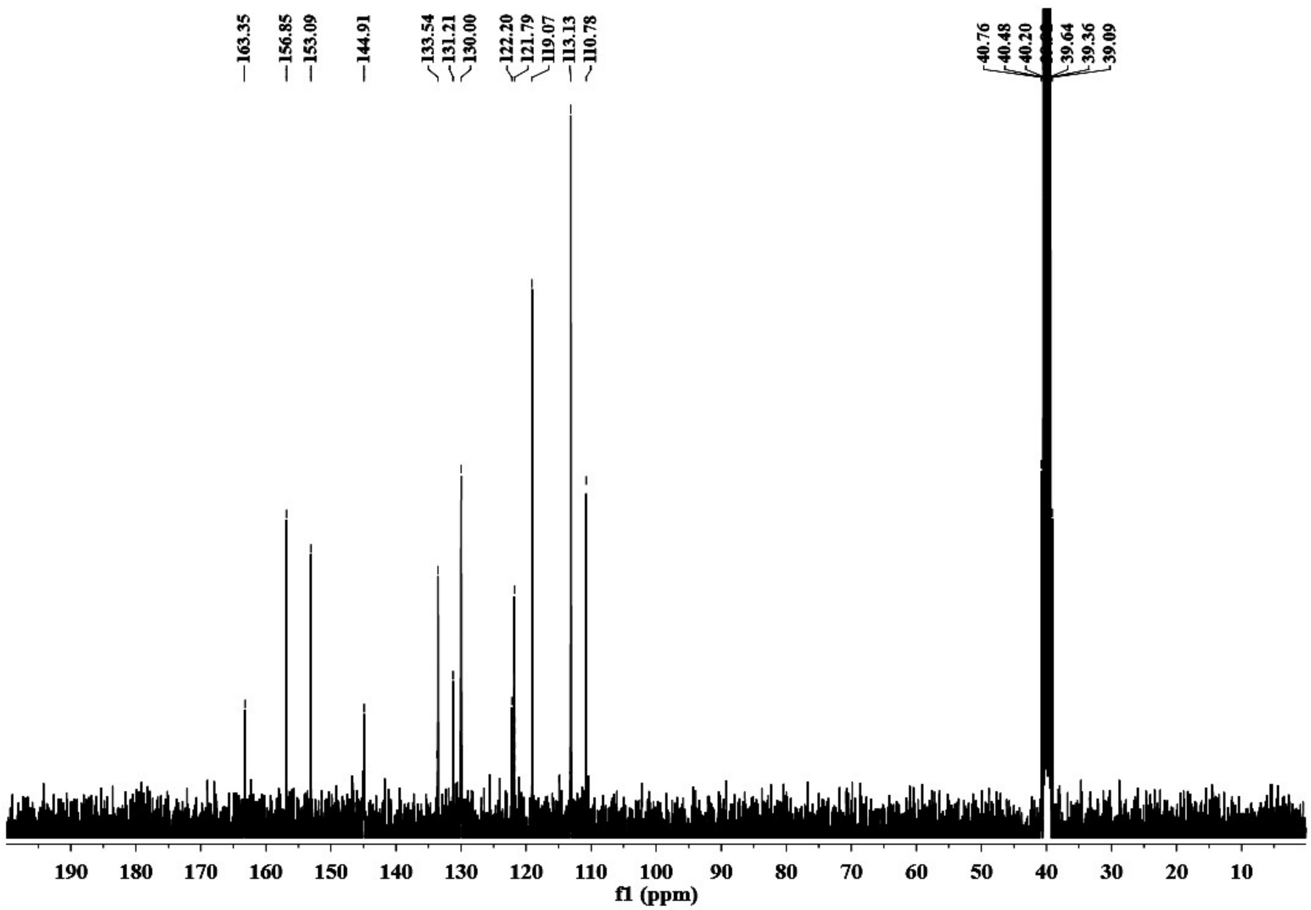

Fig. S4. ^{13}C-NMR spectrum of H_2L in DMSO-d_6

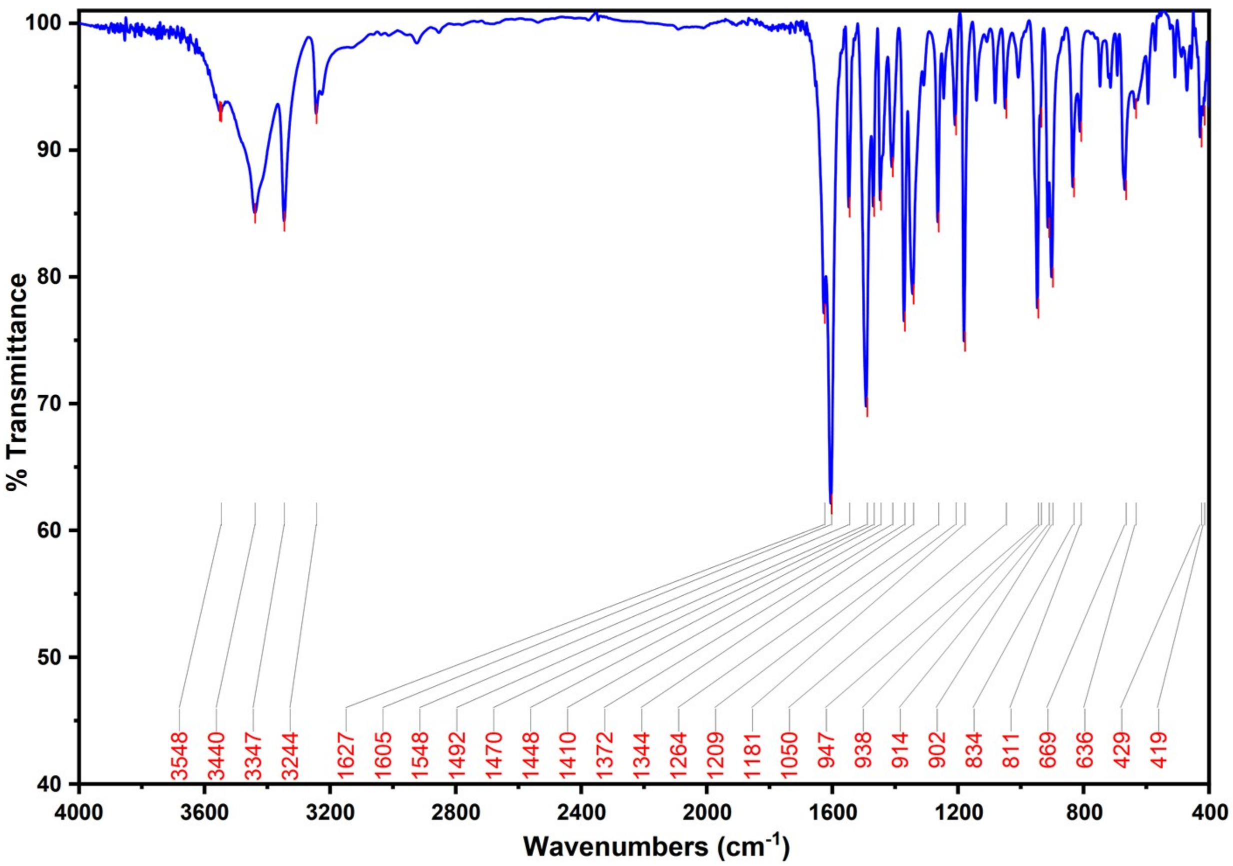

Fig. S5. FT-IR spectrum of compound **1**

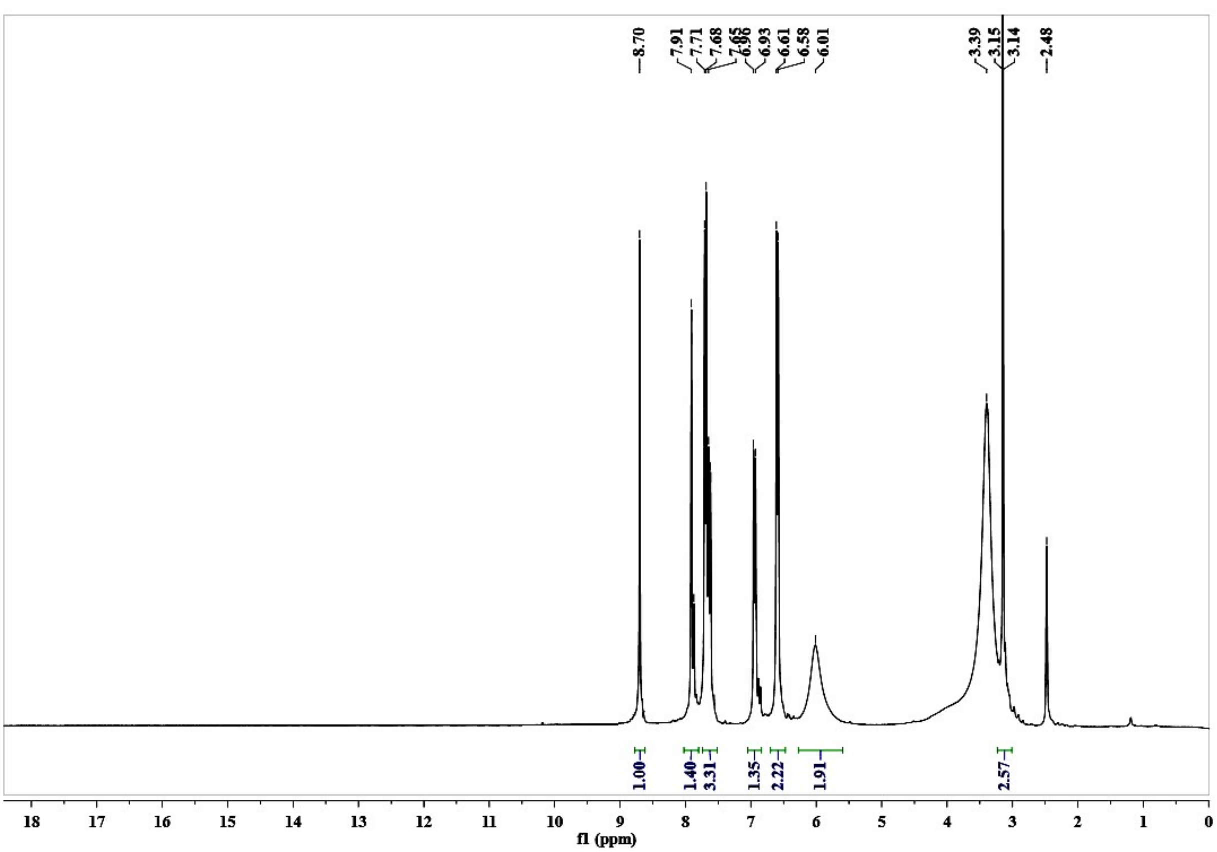

Fig. S6. ^{1}H-NMR spectrum of compound **1** in DMSO-d_6

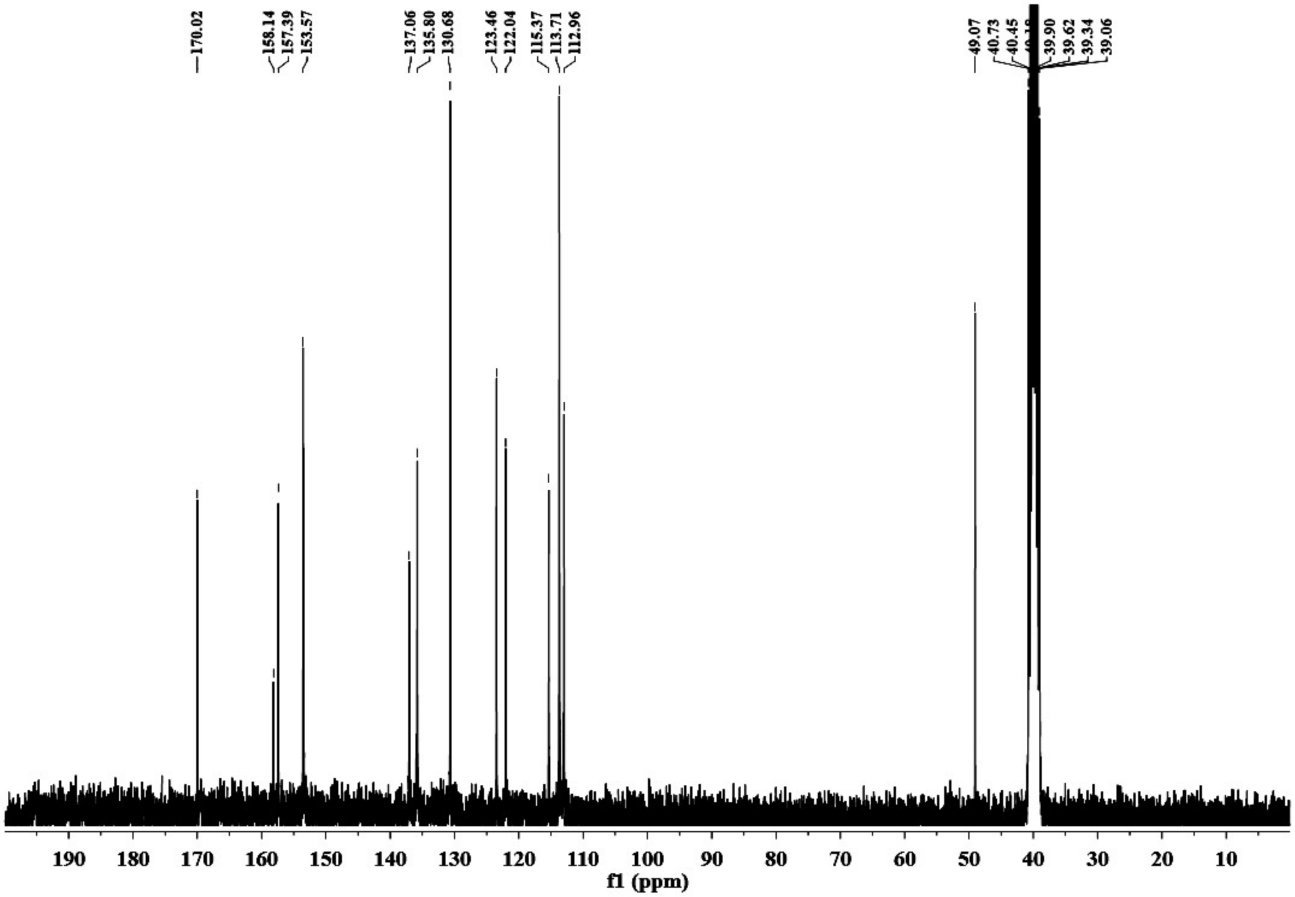

Fig. S7. ^{13}C-NMR spectrum of compound **1** in DMSO-d_6

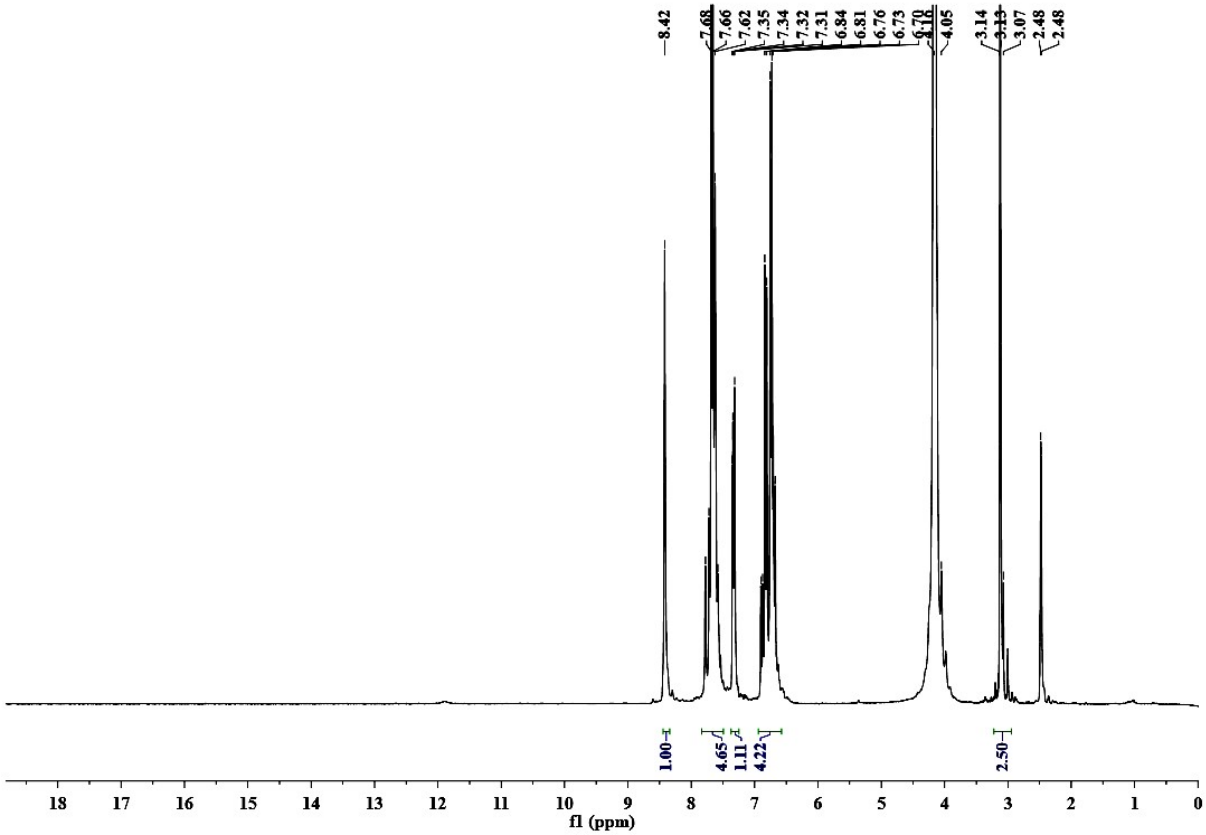

Fig. S8. ^{1}H-NMR spectrum of compound **1** in DMSO-d_6 + D_2O

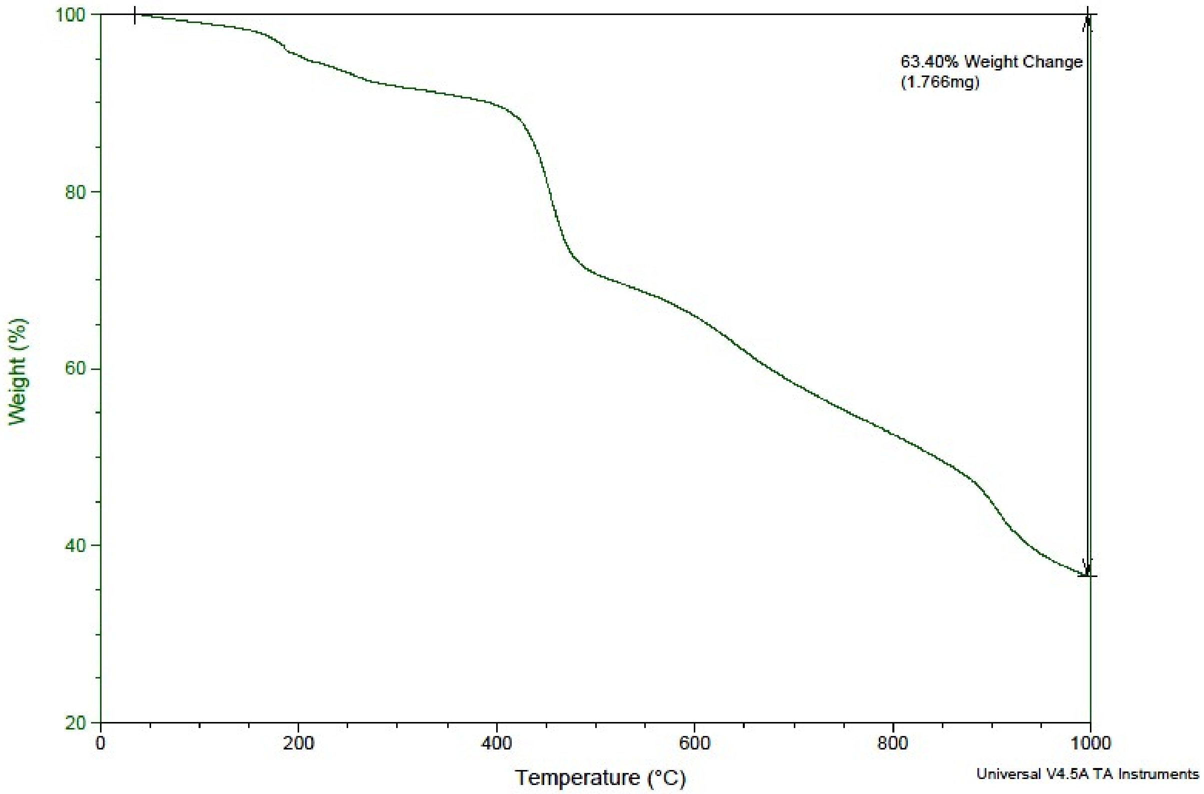

Fig. S9. TGA diagram of compound **1**

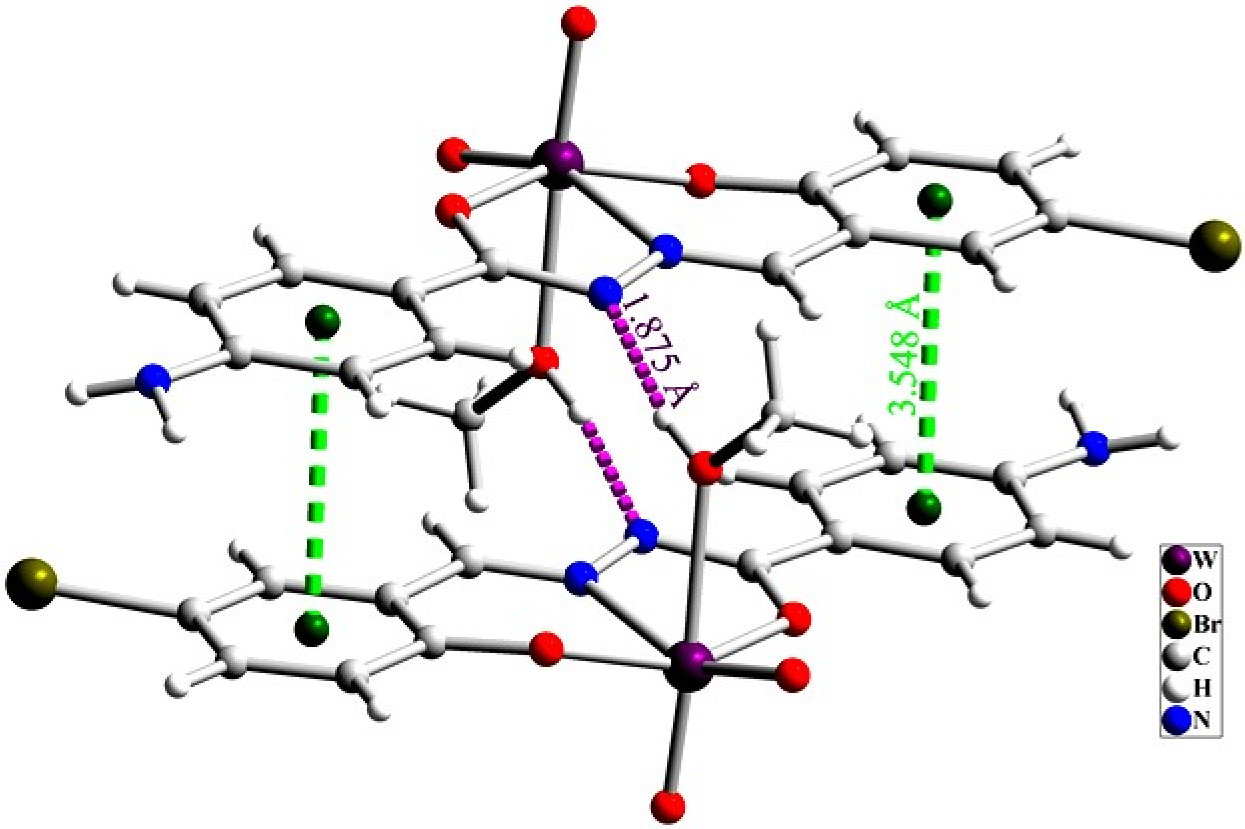

Fig. S10. Pseudo-dinuclear molecules obtained by intermolecular interactions in the crystal structure of **1**

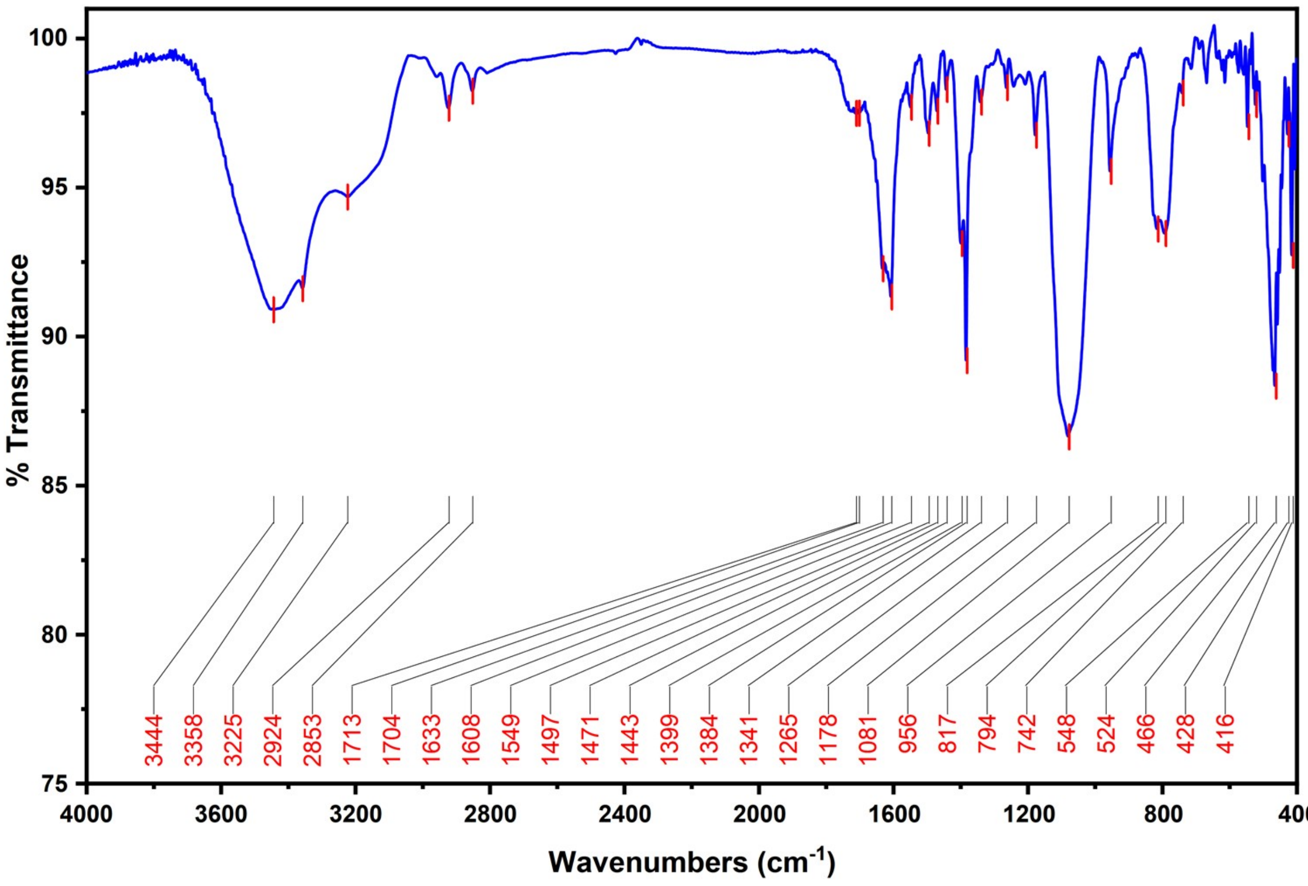

Fig. S11. FT-IR spectrum of supported catalyst

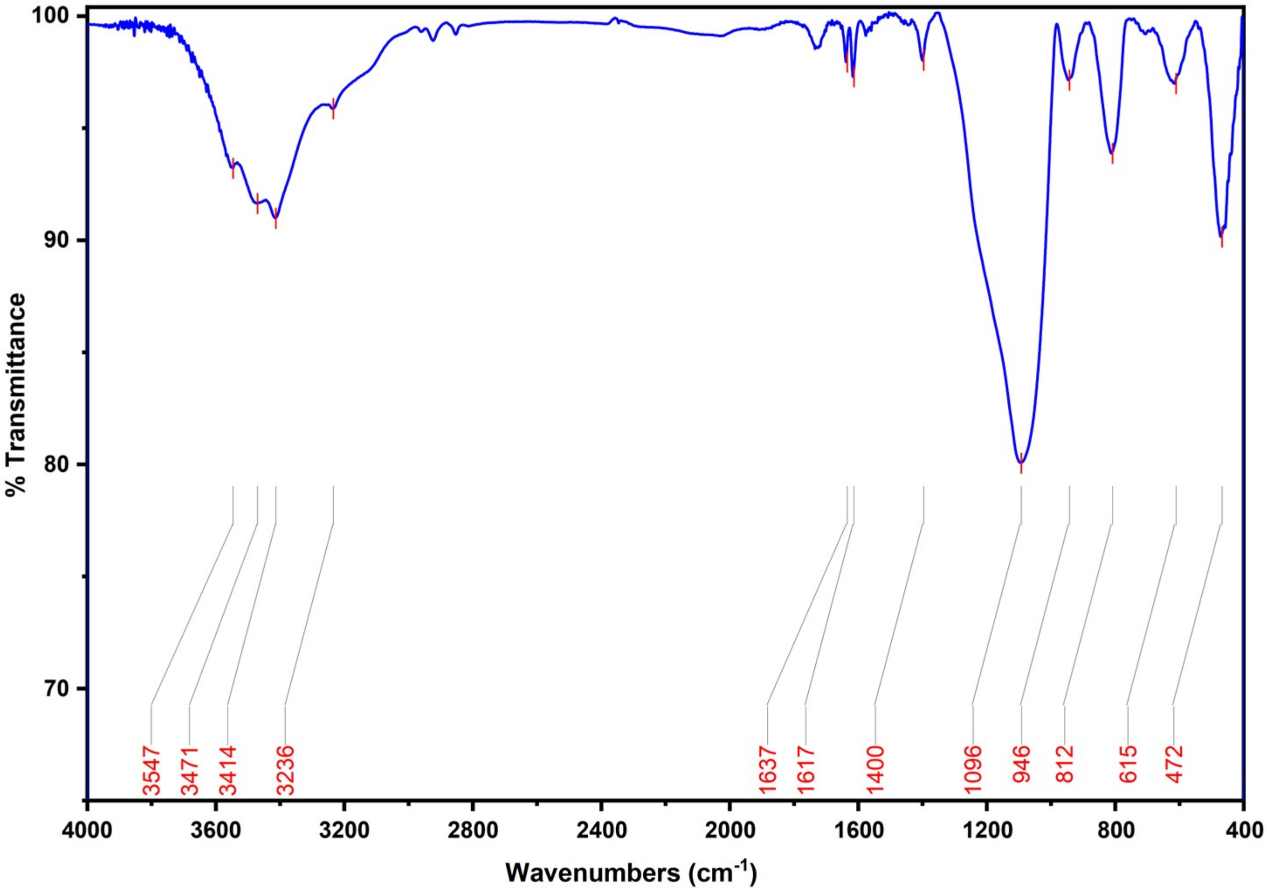

Fig. S12. FT-IR spectrum of propionyl chloride-functionalized silica gel

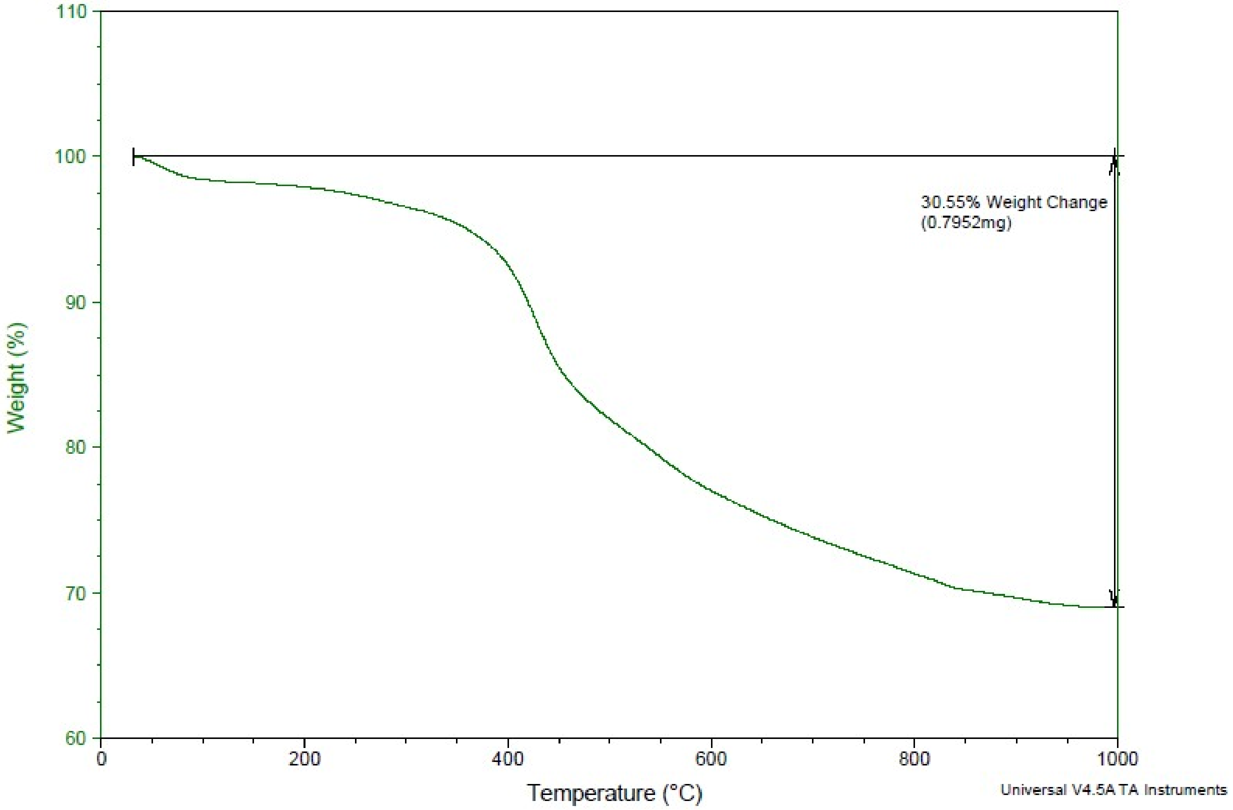

Fig. S13. TGA diagram of supported catalyst

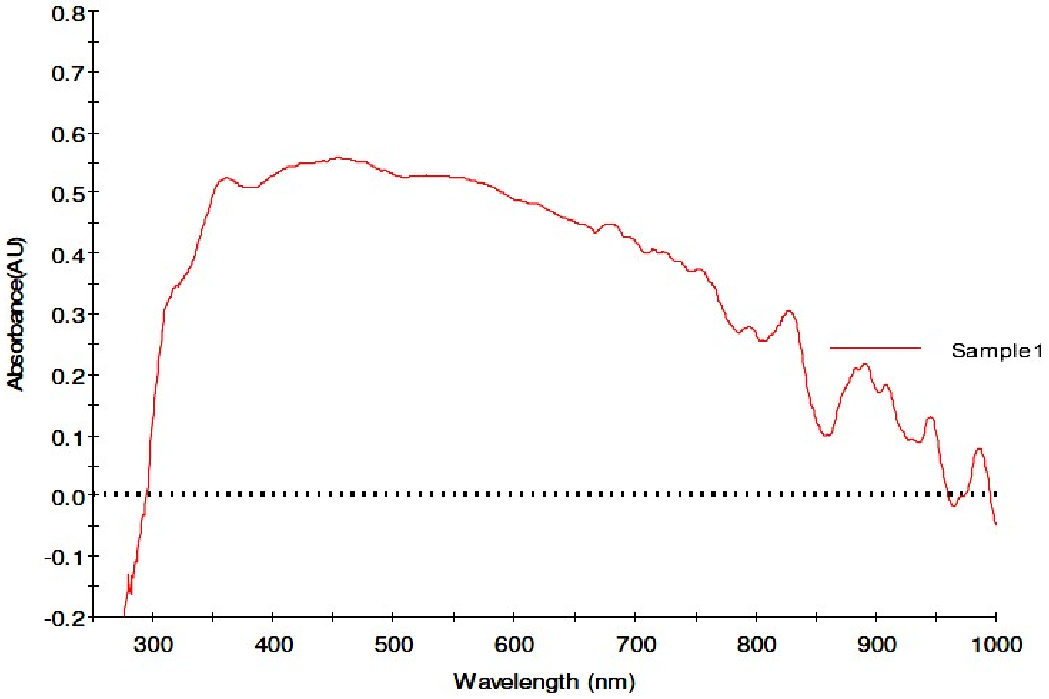

Fig. S14. Difusse-reflectance spectrum of the supported catalyst

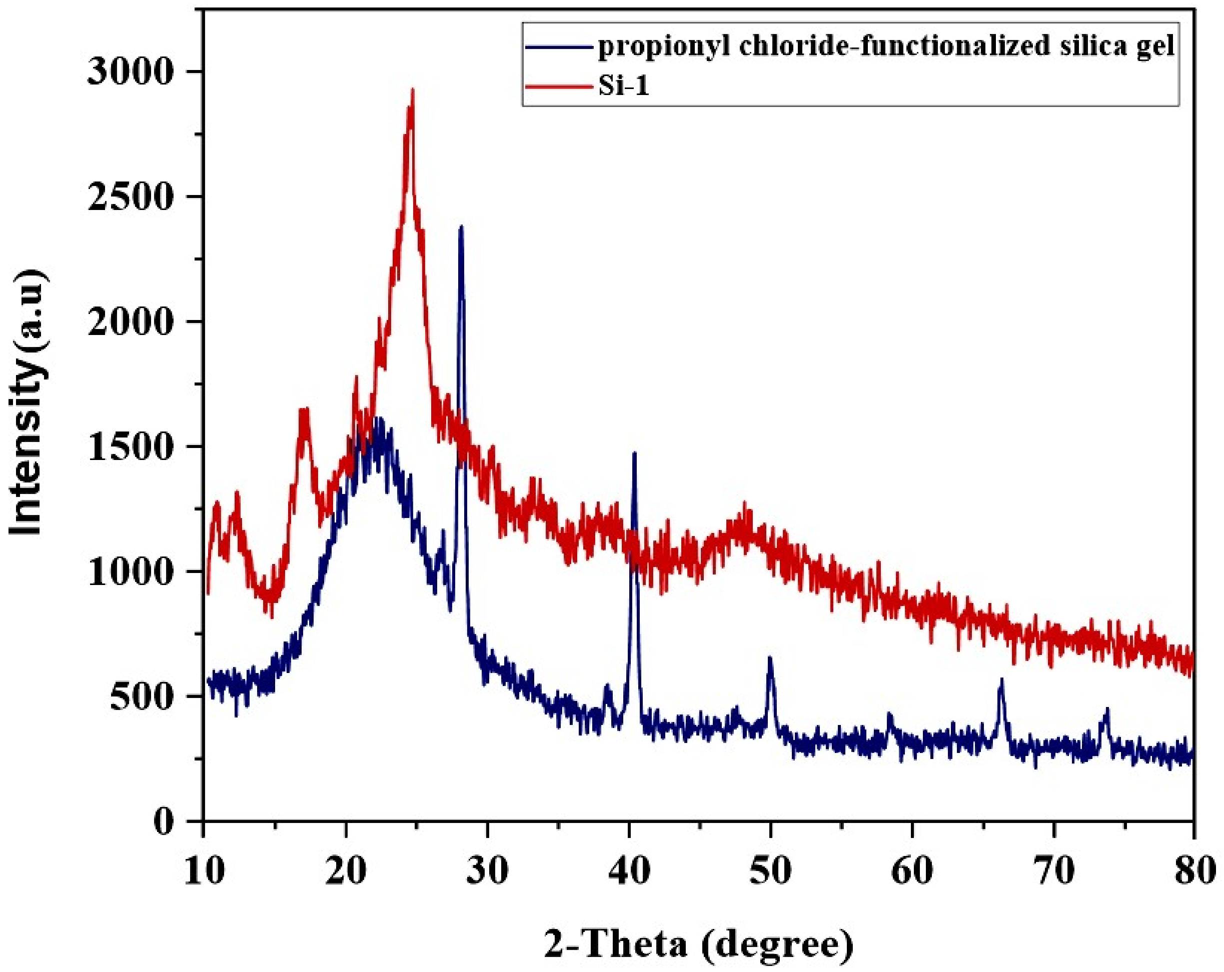

Fig. S15. XRD pattern of propionyl chloride-functionalized silica gel and Si-1

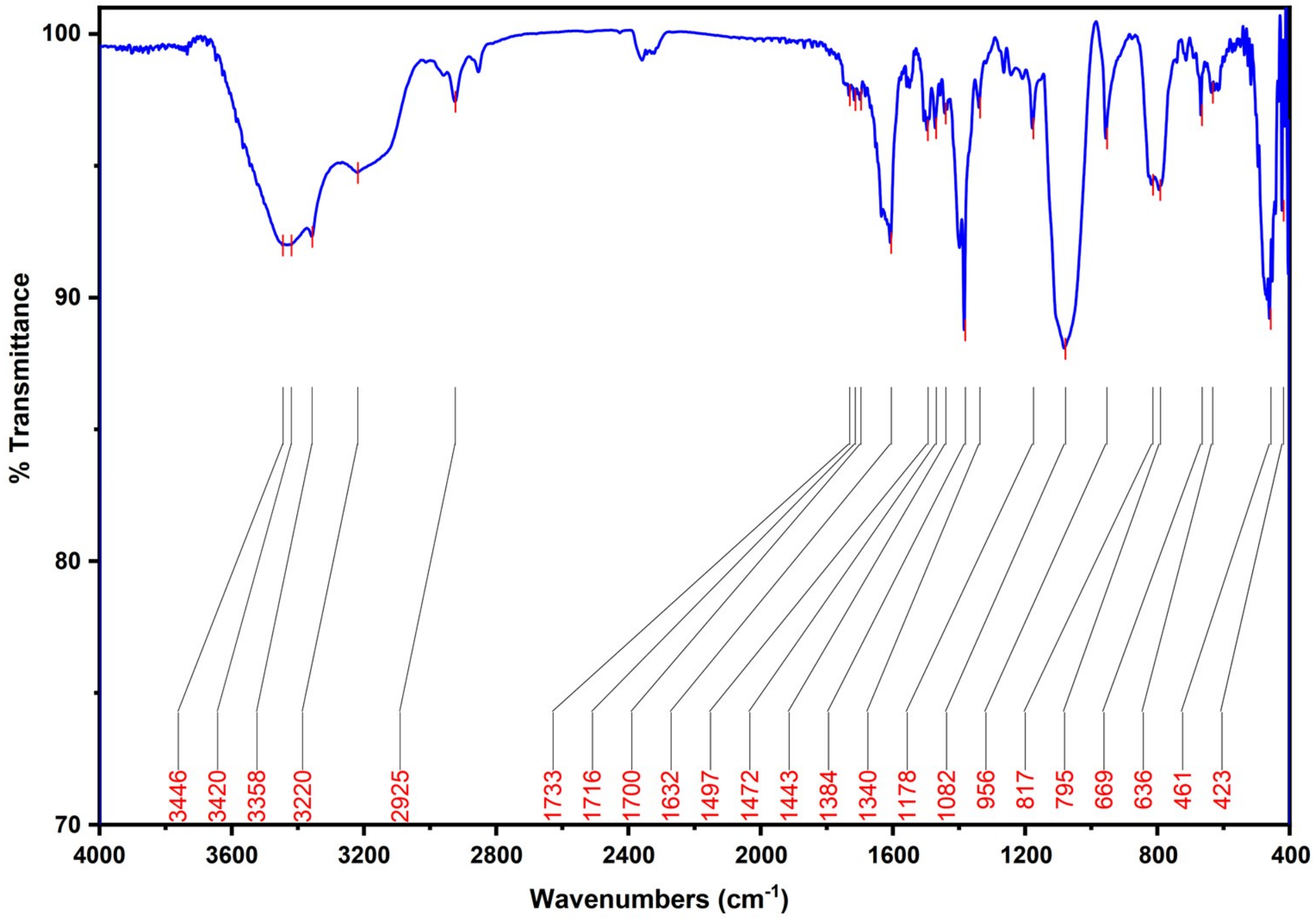

Fig. S16. FT-IR spectrum of recovered catalyst

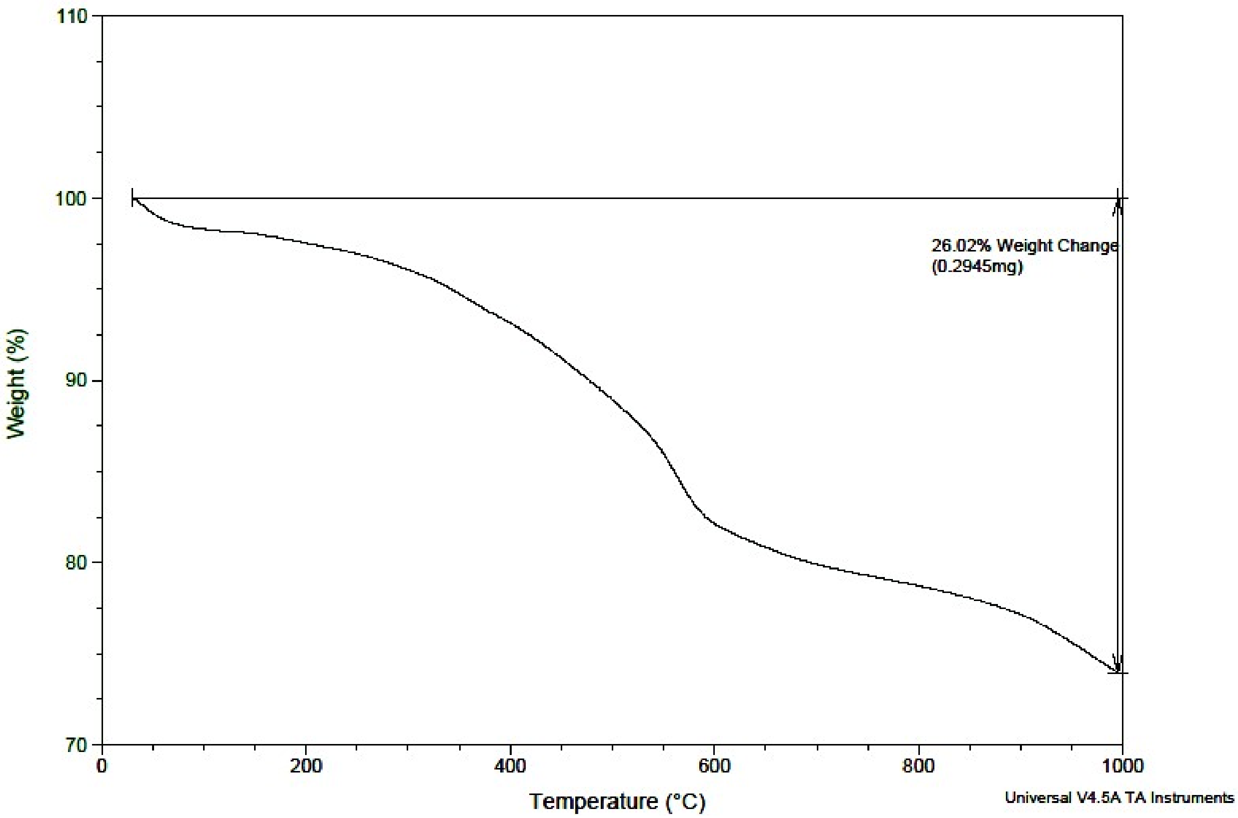

Fig. S17. TGA diagram of recovered catalyst

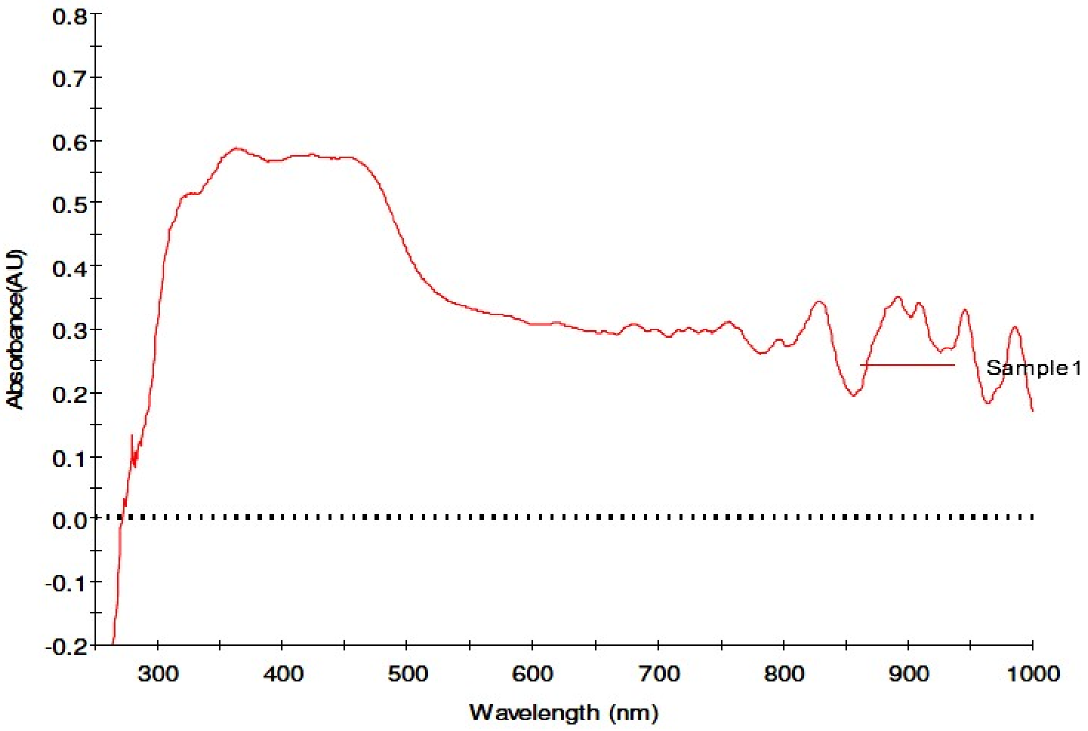

Fig. S18. Difusse-reflectance spectrum of the recovered catalyst

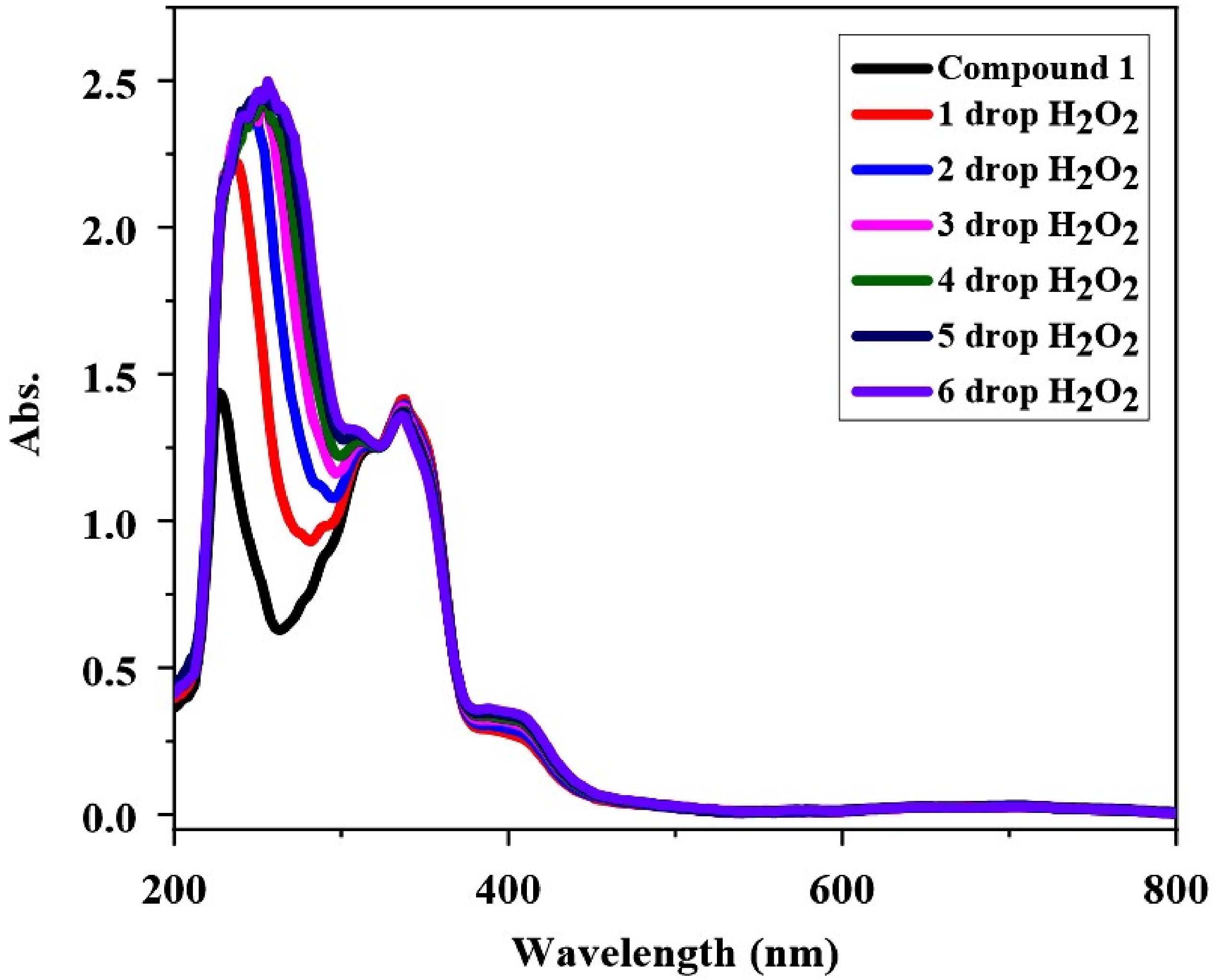

Fig. S19. UV-Vis spectra of compound **1** after addition H_2O_2 (2.5×10^{-5}M) in MeOH
